# Stratigraphy, palaeoenvironments and palaeoecology of the Loch Humphrey Burn lagerstätte and other Mississippian palaeobotanical localities of the Kilpatrick Hills, southwest Scotland

**DOI:** 10.7717/peerj.1700

**Published:** 2016-02-18

**Authors:** Richard M. Bateman, Liadan G. Stevens, Jason Hilton

**Affiliations:** 1 Royal Botanic Gardens Kew, United Kingdom; 2 Earth Sciences, Natural History Museum, London, United Kingdom; 3 School of Geography, Earth and Environmental Sciences, University of Birmingham, Birmingham, United Kingdom

**Keywords:** Anatomical preservation, Bulk geochemistry, Clyde Plateau lavas, Compressions, ICP-AES, Palaeobotany, Palaeoecology, Palaeoenvironments, Palynozones, Permineralisations, Spores, Stratigraphy, Tournaisian, Tuffs, Visean

## Abstract

**Background and Aims.** The largely Mississippian strata of the Kilpatrick Hills, located at the western end of the Scottish Midland Valley, enclose several macrofossil floras that together contain *ca* 21 organ-species of permineralised plants and *ca* 44 organ-species of compressed plants, here estimated to represent 25 whole-plant species (Glenarbuck = nine, Loch Humphrey Burn Lower = 11, Upper = seven). The most significant locality is the internationally important volcanigenic sequence that is reputedly intercalated within the Clyde Plateau Lava Formation at Loch Humphrey Burn, where *ca* 30 m of reworked tuffs and other clastic sediments enclose one of the world’s most important terrestrial lagerstätten of this period. We here explore the palaeoecology and palaeoenvironments of the locality, and elucidate its controversial age.

**Methods.** Repeated re-excavation of key exposures allowed recognition of five main depositional units, differing in thickness from 4 m to 12 m. It also permitted detailed sampling for plant macrofossils and microfossils throughout the succession. Several approaches are integrated to re-assess the taphonomy and preservation of these exceptional plant fossils.

**Key Results.** The deposits are rich in taxonomically diverse miospores and in toto contain at least six well-developed compression floras, together with two beds yielding nodules that enclose well-researched anatomically preserved plants permineralised in calcite. Bulk geochemistry shows that the upper nodules formed by migration of Ca with subordinate Mn and Na. Some phylogenetically important plant fossils recovered in the early 20th century have been traced to their source horizons. Trends in relative proportions of macrofossil and microfossil taxa through the sequence are only moderately congruent, perhaps reflecting the likelihood that microfossils sample the regional rather than the local flora.

**Conclusions.** The Loch Humphrey Burn sequence encompasses a wide range of depositional environments that intercalates high-energy fluvial channels (possibly developed during flash floods in a seasonally arid environment) with lower energy flood plains and a brief lacustrine interval; all yield macrofloras typically dominated by allochthonous pteridosperms. The uppermost unit represents clastic swamps dominated by (hypo)autochthonous lycopsids and ferns s.l., and is tentatively correlated with the entire—reputedly mid-Visean—exposure at nearby Glenarbuck. Other nearby localities with rooted tree-lycopsids appear to have immediately pre-dated the onset of regional volcanism. These interpretations allow revised provenancing and dating of historical collections of key plant fossils. The late Tournaisian date previously attributed on palynological evidence to the lowest unit at Loch Humphrey Burn appears increasingly improbable when our re-appraisal of the macrofloras and microfloras is placed in the context of (a) statistical comparison with other permineralised Mississippian assemblages and (b) recent stratigraphic and geochronologic studies in the region; rather, we ascribe the entire Kilpatrick Hills sequence to the mid-Visean. Stratigraphic and palaeoenvironmental interpretations of the Mississippian rocks of the Kilpatrick Hills have especially profound implications for our understanding of the physical evolution of Scotland during the Variscan orogeny and formation of Pangea.

## Introduction

### Palaeobotany of Scottish Mississippian volcanic terrains

**Figure 1:**
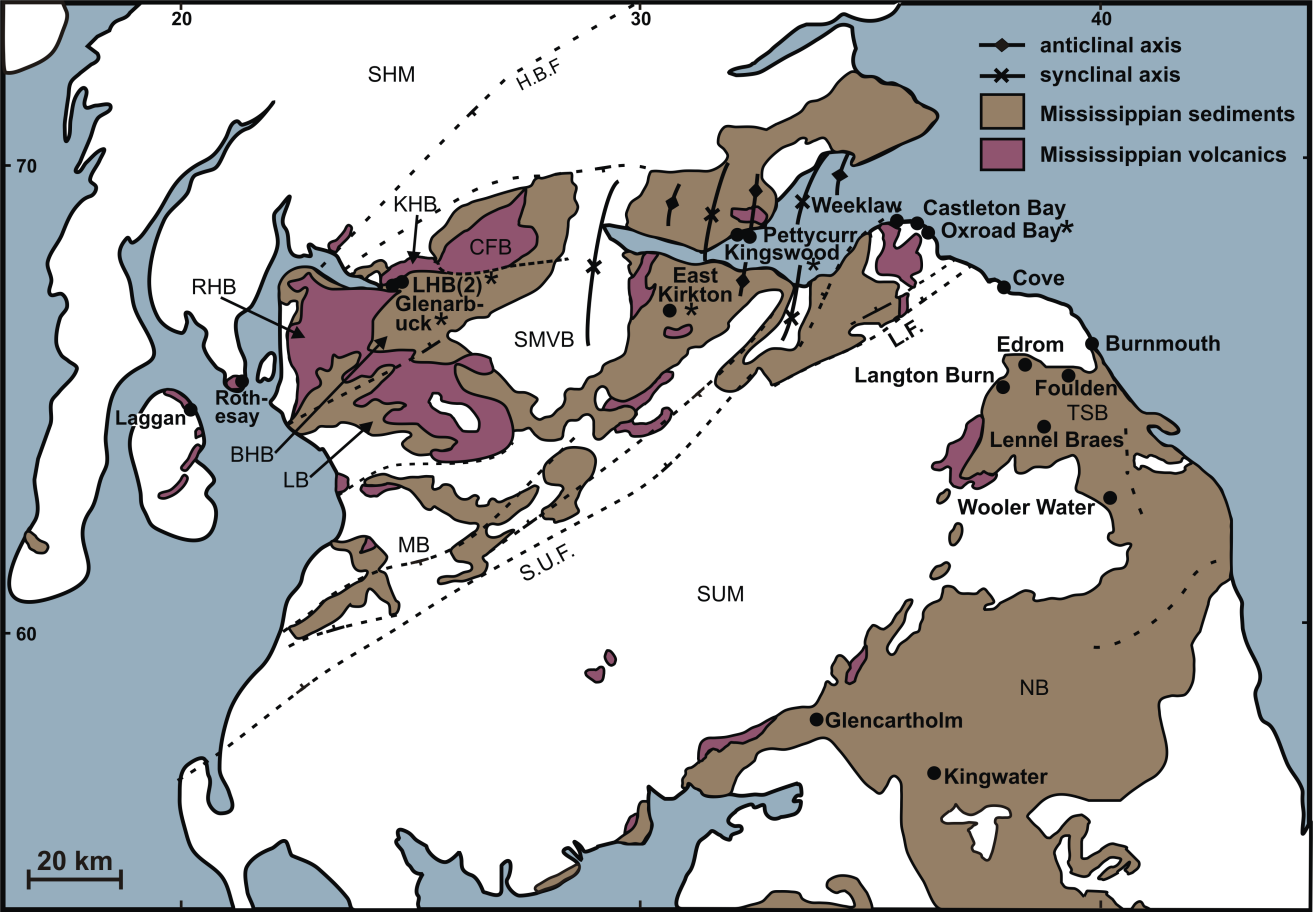
Distribution of permineralised Mississippian floras in northern Britain. Sites discussed in the present study are asterisked. Faults: H.B.F., Highland Boundary Fault; S.U.F., Southern Uplands Fault; L.M., Lammermuir Fault. Highs and lows: SHM, Scottish Highlands Massif; SMVB, Scottish Midland Valley Basin; MB, Mauchine Basin; SUM, Southern Uplands Massif; TSB, Tweed Sub-Basin; NB, Northumberland Basin. Structural units of the Clyde Plateau lavas: LB, Lanarkashire Block; BHB, Beith Hills Block; RHB, Renfrewshire Hills Block; KHB, Kilpatrick Hills Block; CFB, Campsie Fells Block. Information largely derived from Bateman (1991, text-fig. 1) and Monaghan & Parrish (2006, Fig. 1).

The Geological Conservation Review for Palaeozoic palaeobotany (Cleal & Thomas, 1995, Fig. 1.5) revealed two concentrations of internationally important fossil plant localities in the British Isles: dominantly Siluro-Devonian localities along the Welsh Borders, and Devono-Carboniferous localities in and around the Scottish Midland Valley. The best of the Scottish localities occur at either end of the Valley and, due either directly or indirectly to associated volcanicity, preserve plant macrofossils in anatomical detail (Scott, Galtier & Clayton, 1984; Scott, 1990; Scott & Galtier, 1996); they are also intimately associated with stratigraphically diagnostic microfossil assemblages (Clayton et al., 1978). As noted by Cleal & Thomas (1995, p. 113) these deposits “include some of the most important Lower Carboniferous palaeobotanical sites in the world, (notably) the Pettycur Limestone in Fife, the Oxroad Bay Tuff in the Lothians, and the Clyde Plateau Volcanic Formation in Strathclyde” (Fig. 1).

**Figure 2:**
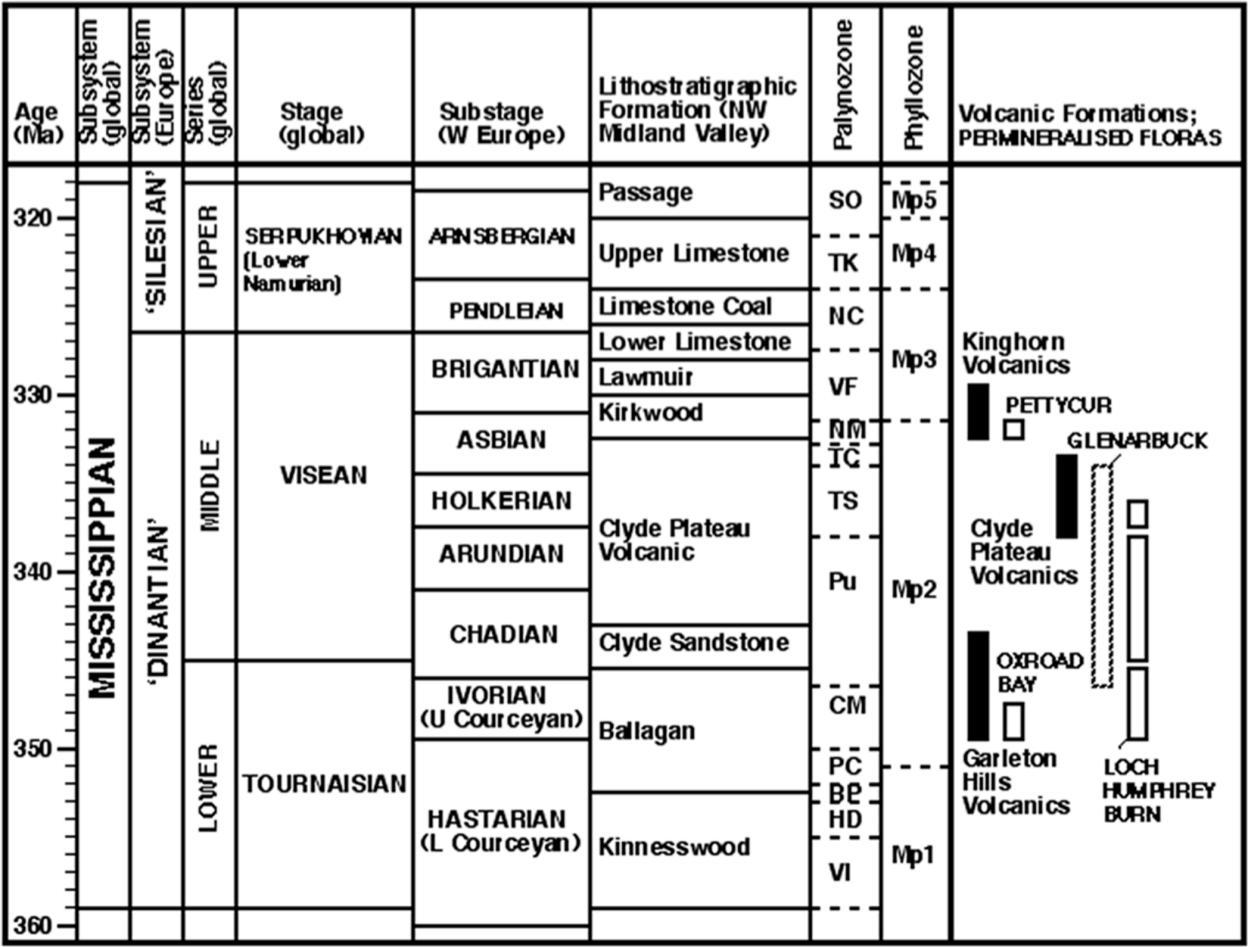
Stratigraphy of the Mississippian subsystem (Carboniferous system) in western Europe. Highlighting plant microfossil and macrofossil biozonations, and providing a context for the volcanic formations and plant-bearing successions that are the focus of this paper (listed right). Largely following Davydov, Wardlaw & Gradstein (2004; see also ICS–IUGS, 2004; Subcommission on Carboniferous Stratigraphy, 2005), but temporally compressing the mid-Visean substages following timescale ‘B’ of Menning et al. (2000) tied to the miospore zones of Riley (1993; see also Clayton, McLean & Owens, 2003). Absolute dates of volcanic formations derived from Monaghan & Pringle (2004) and Monaghan & Parrish (2006); miospore zones of plant-bearing strata derived from Scott, Galtier & Clayton (1984), Scott, Galtier & Clayton (1985) and Scott (1990), and phyllozones modified from Wagner (1984).

These three premium fossiliferous localities, which can legitimately be described as exceptionally preserved ‘lagerstätten’, together span much of the late Tournaisian and Visean (Fig. 2). They were first discovered in 1871, 1930 and 1870, respectively, and each has since been subjected to periodic phases of intense palaeobotanical investigation separated by long intervals of inactivity. All three localities were investigated in the 1980s by AC Scott and colleagues, leading rapidly to publications on Pettycur (e.g., Scott et al., 1986; Rex & Scott, 1987) and Oxroad Bay (e.g., Bateman & Rothwell, 1990; Bateman & Scott, 1990), together with papers describing a Visean succession of near-equal quality at East Kirkton (e.g., Galtier & Scott, 1994). However, thus far, the re-examination of the Clyde Plateau volcanics of the Kilpatrick Hills, north of Dumbarton in Strathclyde, has generated only modest preliminary assessments, published by Scott, Galtier & Clayton (1985) and later Bateman & Cleal (1995a) and Bateman & Cleal (1995b).

This paper presents a detailed account of the plant-bearing localities of the Kilpatrick Hills, focusing on stratigraphical and palaeobotanical information gained from an intensive excavation in 1985 by RMB of the Loch Humphrey Burn locality. This was described by Cleal & Thomas (1995, pp. 155, 164) as “a site of outstanding palaeobotanical importance” and “the most significant Visean site in Europe for understanding the evolutionary history of the early gymnosperms and late progymnosperms.” It is rich in plant organ-species, several of which are presently considered endemic. Although the Mississippian is typically portrayed as a period of evolutionary consolidation, following the major land-plant radiation of the Devonian, it is more accurate to state that the Mississippian is characterised by order- and family-level radiations rather than the preceding Devonian class-level radiations (e.g., DiMichele, Stein & Bateman, 2001).

Relative dates derived from plant macrofossil (Walton, Weir & Leitch, 1938; Smith, 1964) and especially microfossil (Scott, Galtier & Clayton, 1984; Scott, Galtier & Clayton, 1985) assemblages from Loch Humphrey Burn have also played a key (if controversial) role in interpreting stratigraphy and dating volcanicity in the region, which are in turn critical for understanding the much-debated structural development of the Scottish Midland Valley during the late Palaeozoic (reviewed by Browne et al., 1999; Monaghan & Parrish, 2006). The CM palynozone date attributed to the lower part of the succession by Scott, Galtier & Clayton (1984) has had a disproportionately large impact on perceptions of the significance of the locality—not least the supposedly earliest occurrences of several fern families reported from these lower floras by Scott, Galtier & Clayton (1985) and Galtier & Scott (1985).

Thus, the Loch Humphrey Burn site has important implications for our understanding of the reconstruction of Mississippian plants and of their phylogenetic relationships, the palaeoecology and palaeoenvironments of volcanigenic terrains, and the dynamic stratigraphy of Scotland through a critical period in its geological history, namely the Variscan orogeny.

### Physical evolution of the Scottish Midland Valley

The rocks of the Kilpatrick Hills have long been recognised as being dominantly Visean in age (345–327 Ma, Fig. 2: Browne et al., 1999; Read et al., 2002). At that time, Scotland was located close to the southern margin of the Laurasian supercontinent, separated from the Gondwanan supercontinent to the south by the east–west-oriented Rheic Ocean, which was of debatable width (cf. Scotese & McKerrow, 1990). Most tectonic reconstructions place Visean Scotland somewhere between 10°S and the equator, its dominant direction of movement unequivocally being northward.

Reflecting both palaeobotanical evidence (Raymond, Kelley & Lutken, 1989) and oceanic oxygen isotope curves (Veizer et al., 1999), conventional wisdom argues that the mid-Visean (Holkerian) was a period of relatively stable and mesic climate, though it occurred within a longer-term global warming trend. Clayton (1985) used miospore assemblages to infer that Scotland supported a southern subtropical (possibly semi-arid) flora through the Mississippian, though the region lay close to the boundary with a more humid tropical equatorial biome. A largely coincident megafloral unit, encompassing northern Britain and eastern Canada, has been termed the Arcadian subprovince (Raymond, Parker & Parrish, 1985; Raymond, Kelley & Lutken, 1989). More recently, studies of oceanic ‘large bedded cherts’ suggest deepening aridity in the interior of North America during the Visean (Cecil, 2015), humidity reasserting itself only at the beginning of the Serpukhovian (Boucot, Chen & Scotese, 2013). Also, the onset of Gondwanan glaciation has been inferred to approximately coincide with the Asbian–Brigantian boundary, on the basis of late Visean stratigraphy in Fife (Fielding & Frank, 2015).

The structural origin of the Scottish Midland Valley can be traced back to the northward subduction of the Iapetus oceanic plate beneath the Southern Uplands during the early Silurian, and the emplacement of the Southern Uplands accretionary prism during the late Silurian, separated from the more stable Caledonian Uplands by the proto-Midland Valley fore-arc complex. According to Leeder (1987), an aggregate of tectonic platelets that included Spain collided with northern Europe during the Mississippian, causing Variscan orogenesis (and associated rhyolitic volcanism) that was centred on the French Massif Central and peaked during the Visean. Extensional tectonics in the Northern Foreland of northern England and southern Scotland generated the ‘Northern Megabasin’: a graben/half-graben complex of west–east elongated blocks and troughs separated by fault-controlled hinge belts and undergoing differential subsidence (cf. Grayson & Oldham, 1987; Leeder, 1987). This structural activity was associated with widespread pulses of intra-continental basalts (Upton et al., 2004); they peaked in the mid-late Visean (Monaghan & Parrish, 2006) and probably reflected upwelling under the extending back-arc.

However, interpretations more recent than Leeder (1987) have given greater emphasis to either sinistral (Coward, 1993; Rippon, Read & Park, 1996; Read et al., 2002) or dextral (Hooper, 2004) strike-slip activity. For example, the detailed chronology of Monaghan & Parrish (2006 Figs. 7, 9; cf. Read et al., 2002) perceived a shift from polyphase extensional to strike-slip activity in the early-mid Visean, rapidly generating the widely acknowledged north–south oriented anticlinal and synclinal axes in the eastern half of the Valley. They considered the half-graben and horst block on the western half of the Midland Valley to have developed later (Serpukhovian–lower Moscovian, equivalent to Namurian–Westphalian).

### Mississippian geology of the Kilpatrick Hills

Volcanic influence was much greater on sedimentation in the Glasgow Basin, immediately east of the Kilpatrick Hills, than in the depositional basins further east (Fig. 1). Up to 1 km of dominantly basaltic lavas and subordinate tuffs and agglomerates of the Clyde Plateau Volcanic Formation (Paterson, Hall & Stephenson, 1990) capped the rapidly alternating, often cyclical successions of sandstones, shales, marls and cementstones that constitute the Ivorian–Chadian Clyde Sandstone Formation (Owens, 1980a; Browne et al., 1999). These volcanics approximate the Tournaisian–Visean boundary (set at 345 ± 2 Ma: Davydov, Wardlaw & Gradstein, 2004). Extrusion of these extensive lavas eliminated marine influence into the Midland Valley from the west while an equally extensive fluvio-deltaic system prograded south-westward into the fault-bounded Midland Valley (Leeder, 1987).

Understanding of the stratigraphy of the Clyde Plateau has been informed strongly by two contrasting sources of dates: absolute dates provided by Ar/Ar (Monaghan & Pringle, 2004; Monaghan & Browne, 2010) and U–Pb (Monaghan & Parrish, 2006) radiometric analyses of lavas, and relative dates provided by miospore biozones (Scott, Galtier & Clayton, 1984; Riley, 1993; Clayton, McLean & Owens, 2003). Inevitably, both categories of data were set in the context of an evolving perception of regional lithostratigraphy (e.g., Browne et al., 1999). The BGS Loch Humphrey Burn borehole (Owens, 1980a), located 800 m west of the fossil plant-bearing outcrops, and other more recent BGS summaries of the local geology (BGS, 1993; Hall, Browne & Forsyth, 1998), together provide a valuable—but nonetheless equivocal—stratigraphic yardstick.

High-precision radiometric analyses have recently been performed. Monaghan & Browne (2010) dated at 338 ± 18 Ma one sample from within the Clyde Plateau Lavas of the Kilpatrick Hills block, and Monaghan & Parrish (2006) dated at 335 ± 2 Ma the Clyde Plateau lavas of the Renfrewshire Hills Block, immediately southwest of (and commonly correlated with: Stephenson et al., 2003) the Kilpatrick Hills Block (Fig. 1). Following the ‘B’ time-scale of Menning et al. (2000) the more precise Refrewshire date is late Holkerian, and hence falls within the TS palynozone (Fig. 2). Monaghan & Parrish (2006) also inferred a surprisingly brief period of volcanic extrusion, which was apparently confined to the Holkerian and earliest Asbian (mid-Visean). Thus, by definition, it interrupted the Chadian–early Asbian unconformity that is supposed to characterise deposits across much of the Midland Valley (Read et al., 2002). Several associated volcanic necks straddle the Clyde estuary, forming a broad band oriented WSW–ENE, and concentrated in areas of maximal crustal thinning (Francis, 1983).

**Figure 3:**
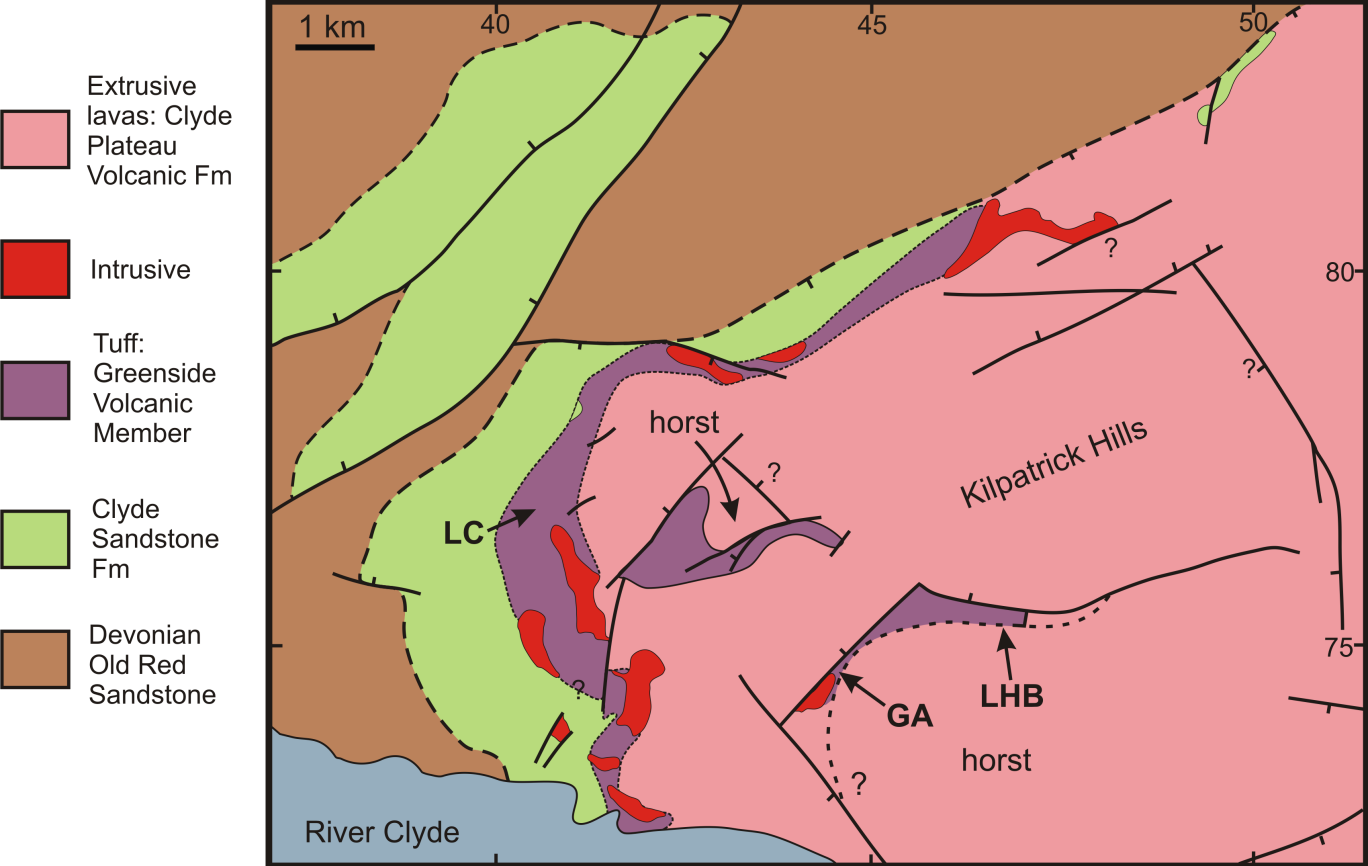
Spatial relationships of the Mississippian plant-bearing localities to the solid geology and faulting in the western half of Kilpatrick Hills. LHB, Loch Humphrey Burn; GA, Glenarbuck; LC, Lang Craigs.

RM Bateman (1986, unpublished data) interpreted the reworked tuffs and other dominantly fluvial deposits that characterise the Glenarbuck and Loch Humphrey Burn sequences as evidence that they were transitional between the Clyde Sandstone sediments and the fully expressed volcanic activity of the Clyde Plateau Volcanic Formation. He invoked considerable vertical displacement of the small, fault-bounded, horst-like blocks evident in the local geology (Fig. 3) to explain the current, relatively high stratigraphic position of the palaeobotanical sites (a similar view was implied by Scott & Galtier’s (1996) description of the plant-bearing deposits as “a pre-Clyde Plateau Lava series”). However, subsequent correlation with the Loch Humphrey borehole suggests that the Glenarbuck and Loch Humphrey Burn sequences constitute a sedimentary body that is intercalated *within* the Clyde Plateau lavas and located well above their base; this body of rock has been formally described as the Greenside Volcaniclastic Member (BGS, 1993; Hall, Browne & Forsyth, 1998). Clarifying the nature of this controversial member constitutes one of the main foci of this paper (‘Stratigraphy and palaeoecology of the Kilpatrick Hills floras’).

### History of palaeobotanical exploration in the Kilpatrick Hills

Plant-bearing localities were first recorded in the complex volcanigenic sequences of the Kilpatrick Hills in about 1870. Field surveyors operating on behalf of the British Geological Survey first located the plant-bearing locality east of a waterfall in Glenarbuck Burn [NS452748] (Smith, 1960). A permineralised specimen of the lycopsid rootstock *Stigmaria* was collected at the locality in 1872, and the presence of this organ-genus was soon reported in a general description of the area by Young (1873).

The classic site for both permineralised and compression floras immediately south of Loch Humphrey Burn [NS468754], 1.3 km ENE of Glenarbuck, was probably discovered by the British Geological Survey at about the same time as Glenarbuck, although the earliest documentary record is a letter that accompanied specimens from Loch Humphrey Burn sent to Robert Kidston by James Bennie in 1886 (Smith, 1960; Scott, Galtier & Clayton, 1984). Much of its subsequent exploration centred upon John Walton at the University of Glasgow (Braid, 1973; Liston & Sanders, 2005).

Early field-workers exploring the Kilpatrick Hills (e.g., Currie, 1865; Young, 1873; Brown, 1935) briefly mentioned other fossil localities along burns to the south and southwest of Glenarbuck, but little subsequent attention has been paid to these sites, or to numerous other exposures of the sub-laval, potentially fossiliferous sediments in the area (Fig. 3). There remain considerable opportunities for the discovery of fossiliferous localities in the vicinity. To cite a relatively modest example, Bateman’s survey in 1985 located moderately large (≤30 cm) calcareous nodules containing only the permineralised stigmarian rootlets of rhizomorphic lycopsids in the extensive cliff sections 2.5 km WNW of Glenarbuck at Lang Craigs (Fig. 3; see also ‘Re-survey and re-excavation of Loch Humphrey Burn’).

## Glenarbuck: Overview

([NS452748]; *ca* +300 m asl)

Interestingly, the discovery in the 1870s of the locality at Glenarbuck, better known than that at Lang Craigs, was also based initially on nodules containing only *Stigmaria*; these features can still be located in the upper part of the Glenarbuck sequence (Scott, Galtier & Clayton, 1984). More strongly calcified nodules containing a far more diverse permineralised flora were collected from the area between 1930 and 1935 by Robert Brown, who also redescribed the locality (Brown, 1935). Fossil plants collected by Brown and presented to John Walton were later described by Calder (1935), Lacey (1953), Smith (1960), Smith (1962c) and Smith (1964). Additional taxa were illustrated by Scott, Galtier & Clayton (1984, their Fig. 7), who logged the site but, despite strenuous efforts, were unable to relocate the main horizon of permineralised nodules. Unfortunately, this failure leaves uncertain the precise origin and age of the vast majority of the plant macrofossils recovered from Glenarbuck.

As summarised by Scott, Galtier & Clayton (1984) and Bateman & Cleal (1995b), the 12-m thick Glenarbuck sequence is part of the “Green tuffs and agglomerates” (the lowest member of the Clyde Plateau Lava Formation), underlain by the “Spout of Ballagan Sandstone” of the Cementstone Group and overlain by the basaltic Clyde Plateau Lavas (Hall, 1978). Miospores recovered by Scott, Galtier & Clayton (1984) appeared to belong to the Pu biozone of the lower Visean (Chadian and Arundian stages: Fig. 2). However, the assemblages were of low diversity and poor preservation, thereby adding unwelcome uncertainty to the estimated age of the beds yielding the macrofossil remains. Moreover, recent correlations place the Glenarbuck sequence within, rather than below, the early-formed lavas, as part of the Greenside Member (BGS, 1993; Hall, Browne & Forsyth, 1998; Monaghan & Parrish, 2006).

The sediments consist of siltstones (including reworked volcanigenic material) and shales intercalated with thin, often discontinuous, organic-rich dark grey bands described in previous literature as ‘coals’ (e.g., Young, 1873) but better termed organic shales (in modern parlance, a coal must exceed 60% fixed organic matter: WA DiMichele, pers. comm., 2015). Also present are abundant, apparently *in situ* rootlets delimiting several weakly defined palaeosols. They are probably river channel and flood plain deposits (Scott, Galtier & Clayton, 1984; Bateman & Cleal, 1995b).

Bateman & Cleal (1995b, p. 166) observed that this Visean flora, modest in its species composition but nonetheless yielding several higher plant taxa, “formed a clastic swamp community” that “provides an interesting contrast with the main plant-bearing units found at nearby Loch Humphrey Burn, which contain fluvially-transported (assemblages) dominated by seed-plants and their immediate progymnosperm ancestors”. The following discussion should make clear that, strictly, Glenarbuck offers an interesting comparison with Loch Humphrey Burn rather than providing a direct “contrast.”

## Loch Humphrey Burn: Overview

(A = [NS4676.7537], B = [NS4682.7537]; both = *ca* +300 m asl)

**Figure 4:**
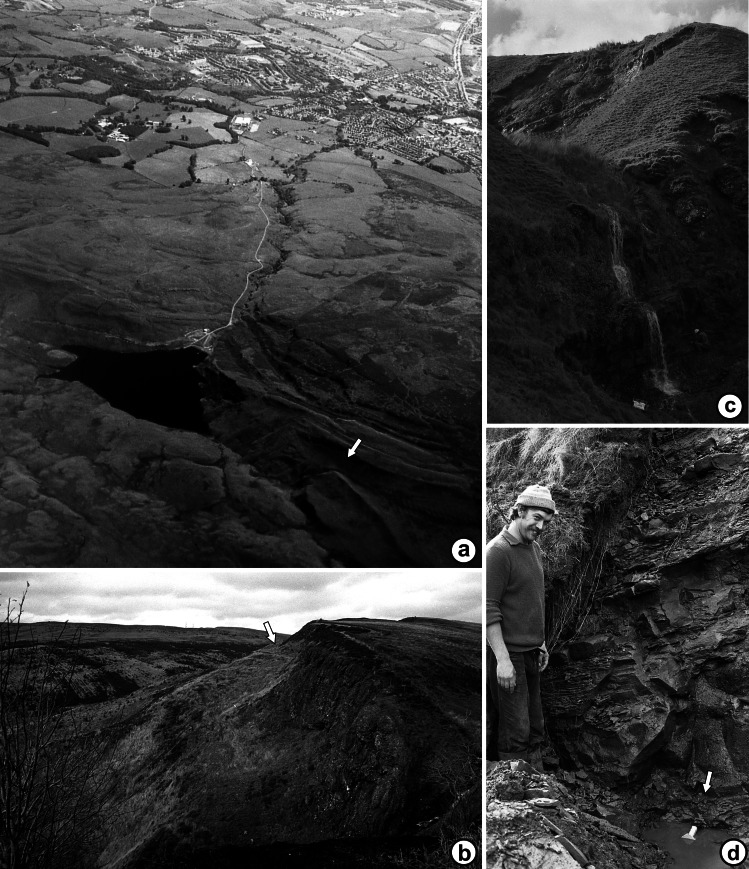
Geomorphological context of the Kilpatrick Hills plant-bearing localities. (A) Aerial view southeastward across the Kilpatrick Hills parallel to Loch Humphrey Burn (Greenside Reservoir in the mid-foreground, Dumbarton in the distance); the classic Loch Humphrey Burn locality is arrowed. (B) View northeastward across the Glenarbuck locality (arrowed). (C) View WSW across the Loch Humphrey Burn locality showing the locations of Exposures A2 (the waterfall), A1 (beyond the waterfall), and B, with human figure for scale. (D) Gordon Barrass standing to the left of the permineralisation-bearing Walton lens of Exposure A1 (arrowed). All images: RMB.

The classic Loch Humphrey Burn Site of Special Scientific Interest occurs where a small northward-flowing tributary of Loch Humphrey Burn has cut a deep (*ca* 18 m) ‘hanging valley’ into a 30 m-high fault scarp of tuffaceous sediments, creating two exposures (Figs. 4A and 4C): Exposure A1 is the steep (*ca* 60°) east-facing bank of the valley (Fig. 4A), and Exposure A2 is a waterfall cascading over the lower part of the scarp, immediately below the valley (Fig. 3C). The locality and sediments are described in greater detail below. It is especially important to unravel the complex, poorly documented history of the site, as Loch Humphrey Burn is actually an aggregate name for three plant-bearing exposures that, collectively, have previously been hypothesised to represent two disjunct depositional phases.

The Loch Humphrey Burn site was probably discovered by the British Geological Survey at about the same time as Glenarbuck, although the earliest documentary record is a letter that accompanied specimens from Loch Humphrey Burn sent to Robert Kidston by James Bennie in 1886 (Smith, 1960; Scott, Galtier & Clayton, 1984). Three putative pteridosperm stems received by Kidston were passed on to DH Scott for formal description (Scott, 1899; Scott, 1902; Scott, 1918; Scott, 1923; Scott, 1924); all three are holotype specimens of the respective organ-species (Table 1A). Unfortunately these fossils now lack any vestige of the enclosing matrix, but they were reputedly extracted from “beds of coarse ash agglomerate on the south side of Loch Humphrey Burn” (Scott, 1924). Kidston also illustrated several specimens from “the escarpment on the south side of Loch Humphrey Burn” in his classic monograph on Carboniferous compressions (Kidston, 1923–1925), emphasising the organ-species diversity of the foliage genera *Rhacopteris*, *Spathulopteris* and *Sphenopteridium* (a full list of compressions recorded from Loch Humphrey Burn is presented in Table 1D).

**Table 1:** List of macrofossil organ-species for Mississippian plant-bearing localities in the Kilpatrick Hills. Higher classification largely follows Stewart & Rothwell (1993), while recognising that some of the higher taxa are paraphyletic rather than monophyletic. Number of specimens known, and any nomenclatural types, are given in square brackets (m, several specimens; S, type species; G, type genus). Citations denote first mention (M) and first illustration (F) in the scientific literature (see also Scott, Galtier & Clayton, 1984; Scott, Galtier & Clayton, 1985; Bateman & Cleal, 1995a; Bateman & Cleal, 1995b).

**Figure 5:**
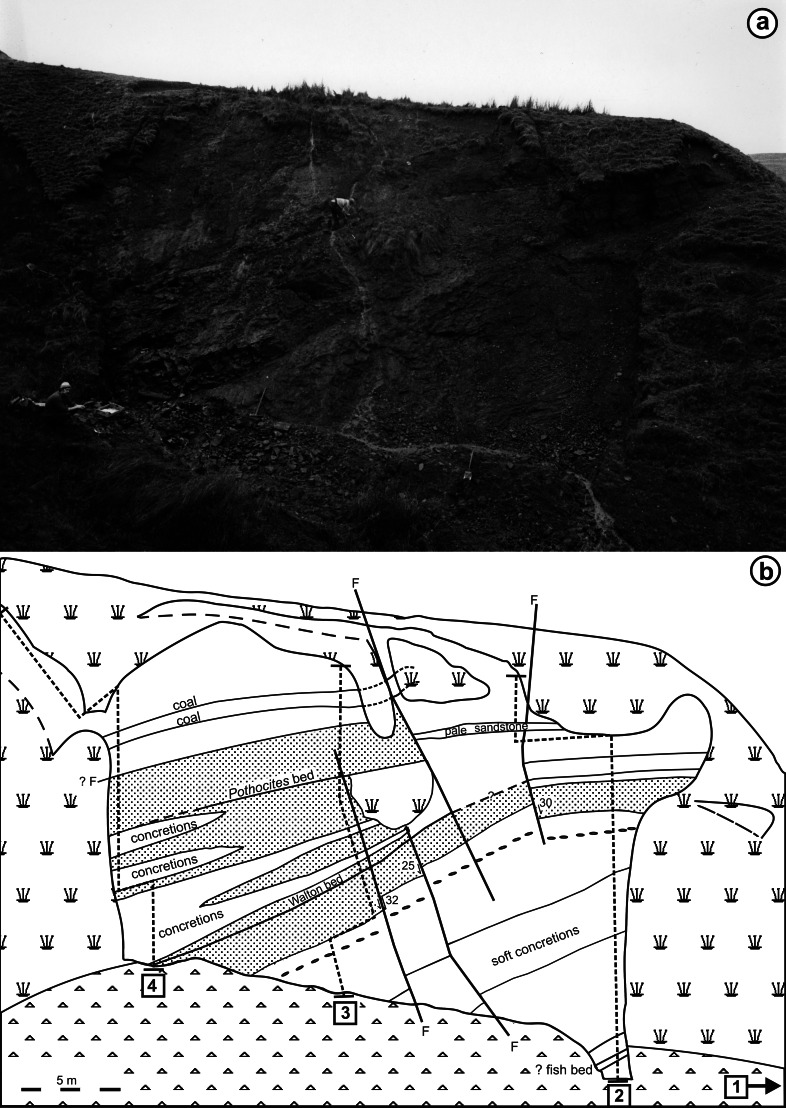
Appearance of the main exposure (A1) at Loch Humphrey Burn immediately following its re-excavation in 1985. (A) Photograph showing Rob Gray (centre) standing on the *Pothocites* bed, while Gordon Barrass (left) is seated immediately to the left of the Walton bed. (B) Interpretation of the exposure highlighting the more prominent beds and minor faults (F), and showing the locations of measured sections 2–4 shown in Fig. 6; section 1 (lower waterfall) is located *ca* 10 m to the northeast (see also Bateman & Cleal, 1995a, their Fig. 5.30).

**Figure 6:**
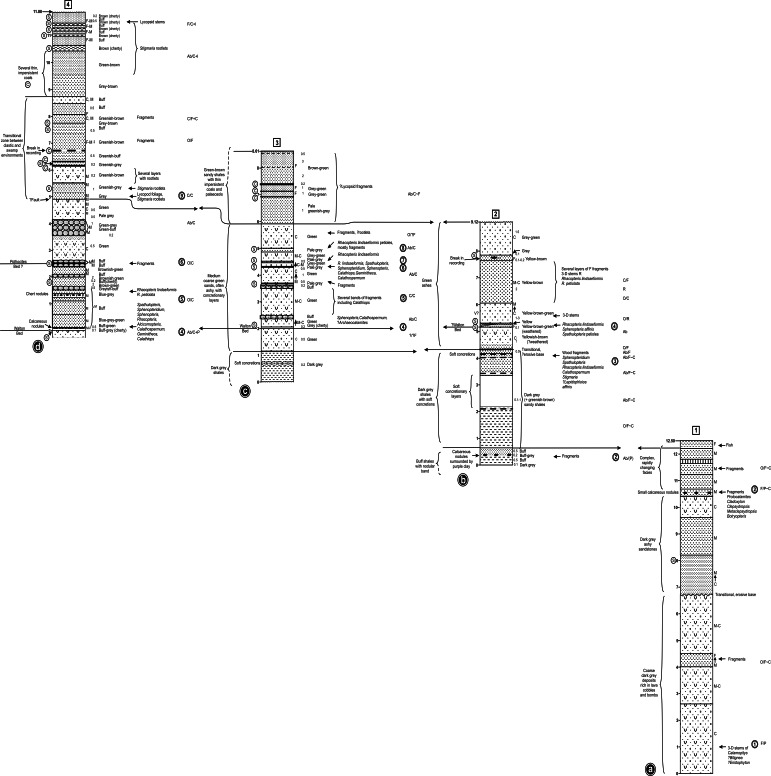
Correlation of three closely-spaced, partially overlapping lithological logs of the Walton site (Exposure A1: Figs. 4 and 5) plus a corresponding log of the lower waterfall site (Exposure A2). Information to the left of each column: circled C indicates a thin black organic shale (‘coal’) band, circled S indicates a horizon sampled for plant macrofossils during the present study. Information to the right of each column: coarseness of sediment; C, coarse; M, medium; F, fine; figures indicate typical lamination intervals in cm (m, massive); background colour of sediment; plant macrofossil content; stratigraphically numbered floras (in double circles); frequency of plant macrofossils (before the slash): Ab, abundant; C, common; F, frequent, O, occasional, R, rare; state of preservation of plant macrofossils (after the slash): P, permineralisation; F, fusain; C, compression; I, impression. Asterisks indicate plant macrofossil remains too fragmentary to allow identification. The logs were prepared by RMB in advance of the internationally accepted Selley format, and cannot readily be revised as the sediments are no longer exposed.

**Figure 7:**
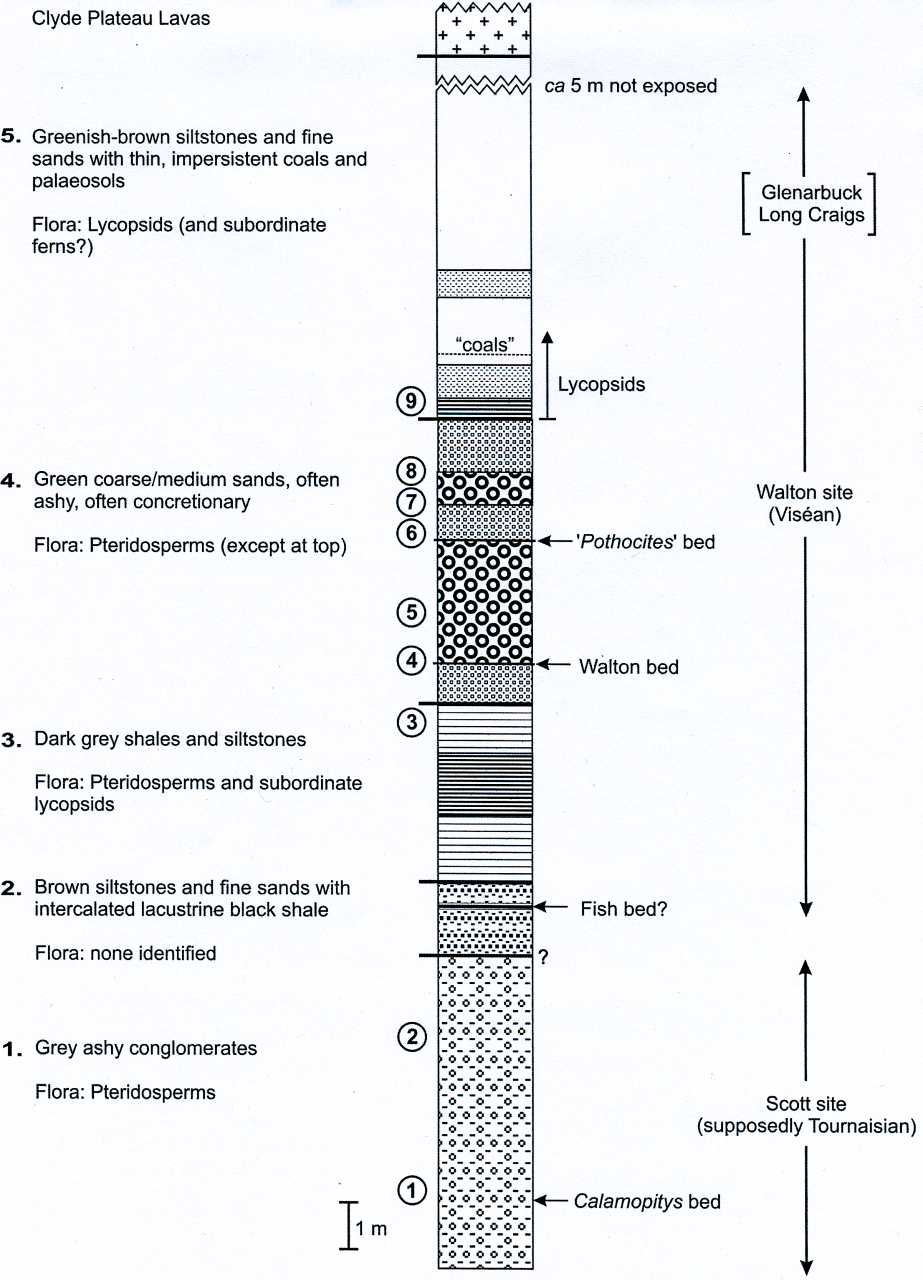
Composite stratigraphy of the Loch Humphrey Burn plant-bearing locality. Information derived from the four overlapping sections logged in Fig. 6, summarising the five-fold stratigraphic subdivision developed in the present study.

**Figure 8:**
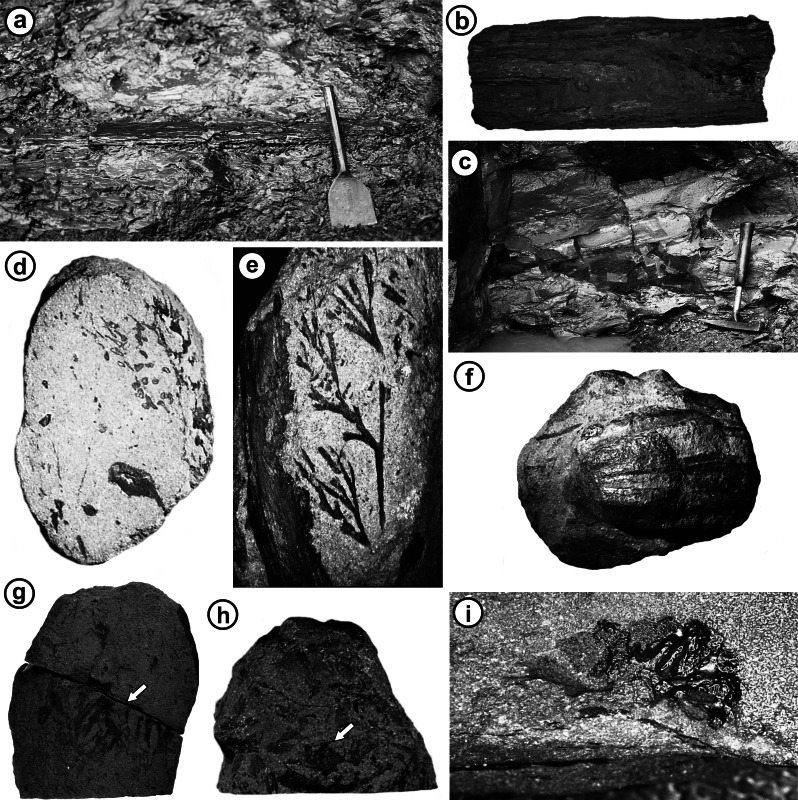
Diversity of plant fossils recovered from Loch Humphrey Burn. (A) Cf. *Calamopitys* branch *in situ* in wood-bearing horizon of Exposure A2 (supposedly late Tournaisian). (B) Portion of the stem shown in (A), following laboratory preparation (17 cm long). (C) The nodule-bearing lens of reworked tuffs in Exposure A1 (the Walton site: Visean). (D–I) Calcareous nodules containing permineralised plants derived from horizon shown in (C). (D) Surface of polished surface of interior of specimen G, showing the multiple lobes and ovules of a cupule pair (arrowed). (E) Nodule with indeterminate petiole (left) and leaf apex of cf. *Sphenopteridium*. (F) Exterior of the ‘megacupule’ *Calathospermum scoticum*. (G) Nodule showing both the compression and permineralised preservation of cf. *Pullaritheca* (arrowed). (H) Nodule showing both the compression and permineralised preservation of *Alcicornopteris* (arrowed). (I) Acid-etched transverse section of the archaeocalamite cone *Protocalamostachys arranensis* (cf. Bateman, 1991).

**Figure 9:**
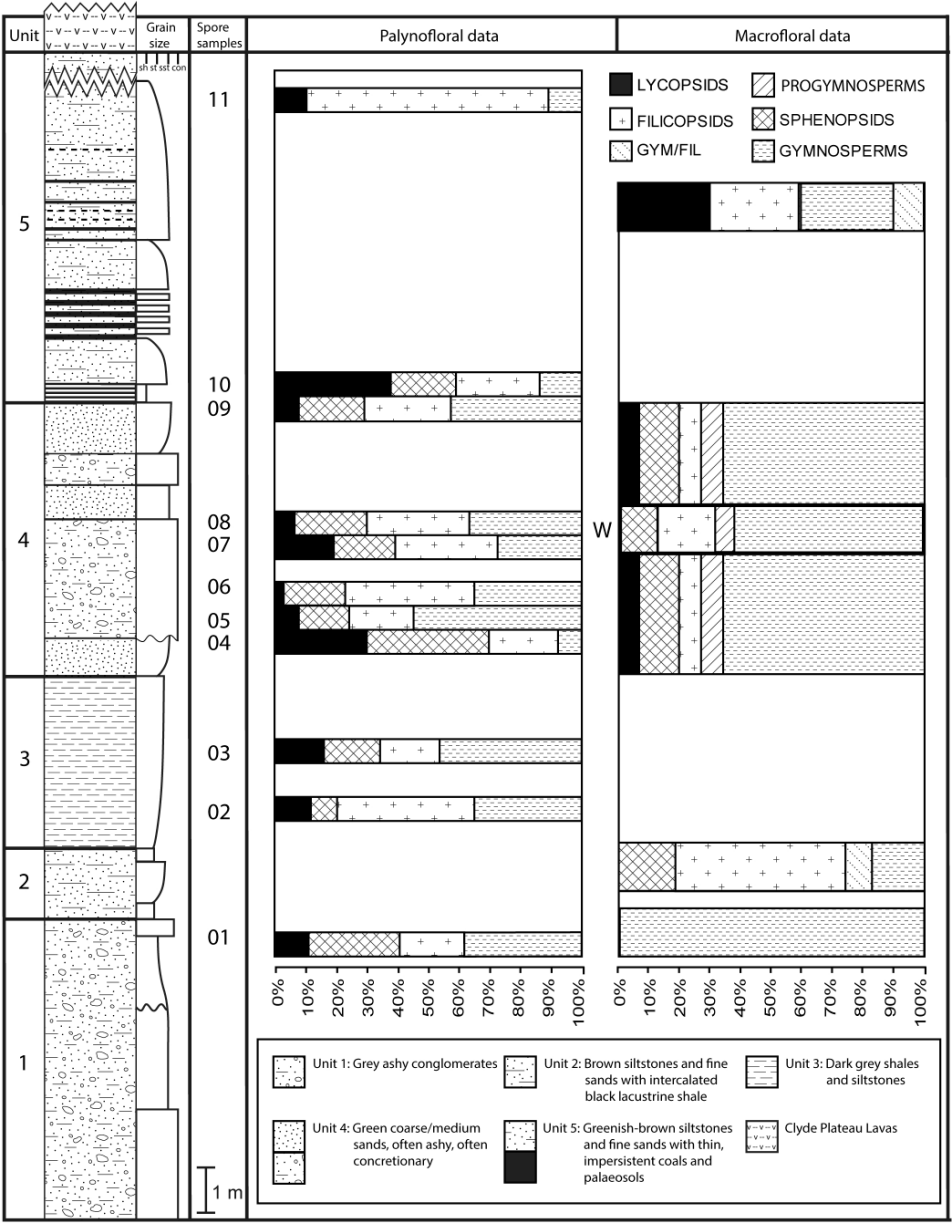
Histograms of the relative proportions of land-plant microfossils (left) and macrofossils (right) in different taxonomic classes. Plotted against a simplified sedimentary log of the Loch Humphrey Burn sequence. Some organ-genera of fern-like foliage cannot be apportioned between ferns and seed-ferns, hence the use of a pteridosperm/fern (‘Gym/Fil’) category in the macrofossil histogram. The well-known Walton Bed is marked W.

**Figure 10:**
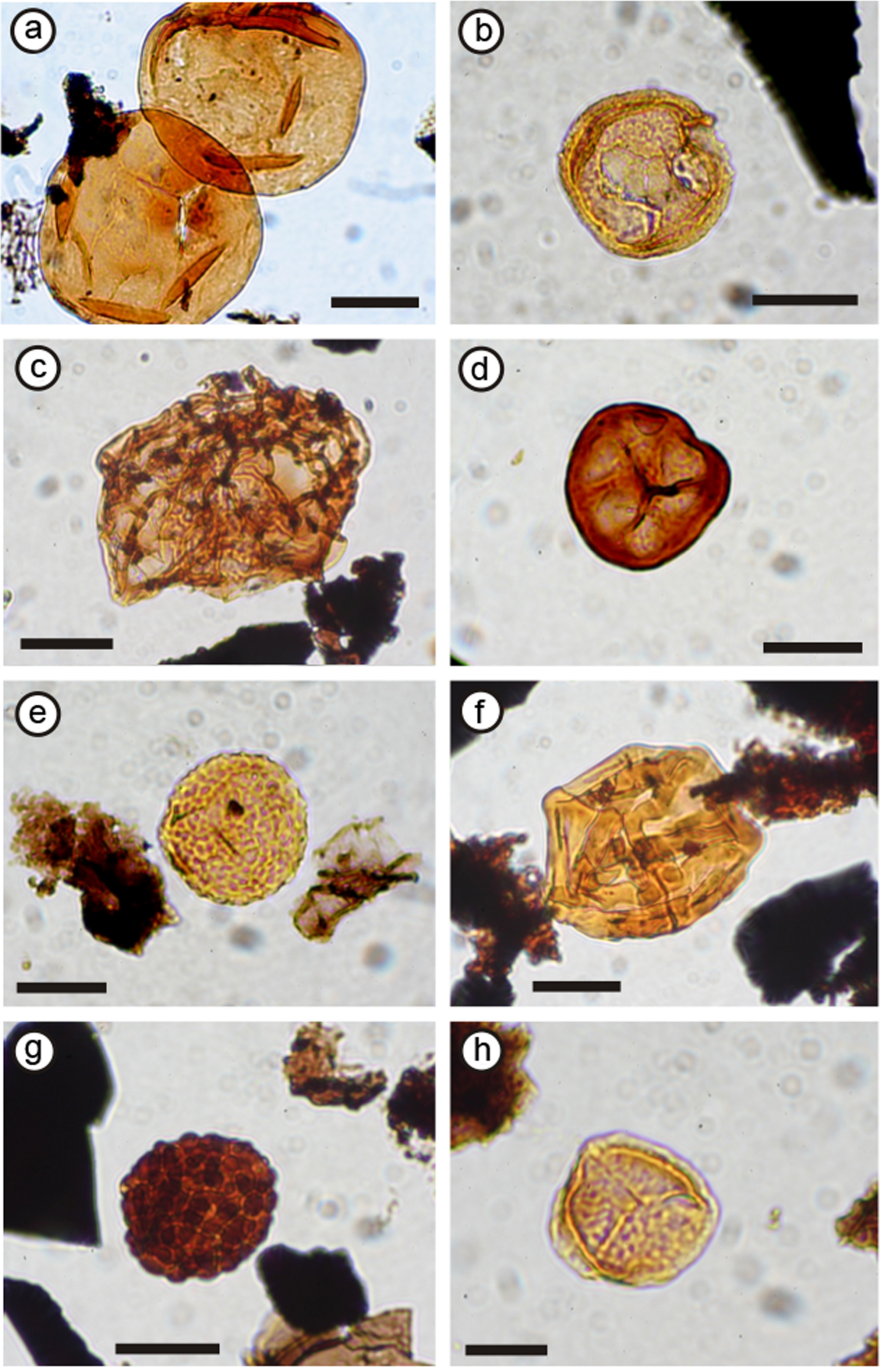
Representative specimens of miospore genera that dominate the microfossil assemblages at Loch Humphrey Burn. (1) *Calamospora* Schopf, Wilson & Bentall, 1944 (sphenopsid), specimen 206, horizon LH-2-15; scale bar = 25 µm. (2) *Cyclogranisporites* Potonié & Kremp, 1954 (filicopsid), specimen 151, horizon LH-4-9; scale bar = 20 µm. (3) *Dictyotriletes* (Naumova) Potonié & Kremp, 1954 (filicopsid), specimen 167, horizon LH-3-13; scale bar = 25 µm. (4) *Knoxisporites* (Potonié & Kremp) Neves 1961 (filicopsid), specimen 244, horizon LH-2-8; scale bar = 20 µm. (5) *Microreticulatisporites* (Knox) Bhardwaj, 1955 (filicopsid), specimen 146, horizon LH-2-15; scale bar = 20 µm. (6) *Reticulatisporites* Ibrahim 1933 (filicopsid), specimen 075, horizon LH-3-13; scale bar = 30 µm. (7) *Verrucosisporites* Ibrahim 1933 (filicopsid), specimen 047, horizon LH-2-5; scale bar = 20 µm. (8) *Lycospora* Schopf, Wilson & Bentall, 1944 (lycopsid), specimen 233, horizon LH-2-15; scale bar = 20 µm.

**Figure 11:**
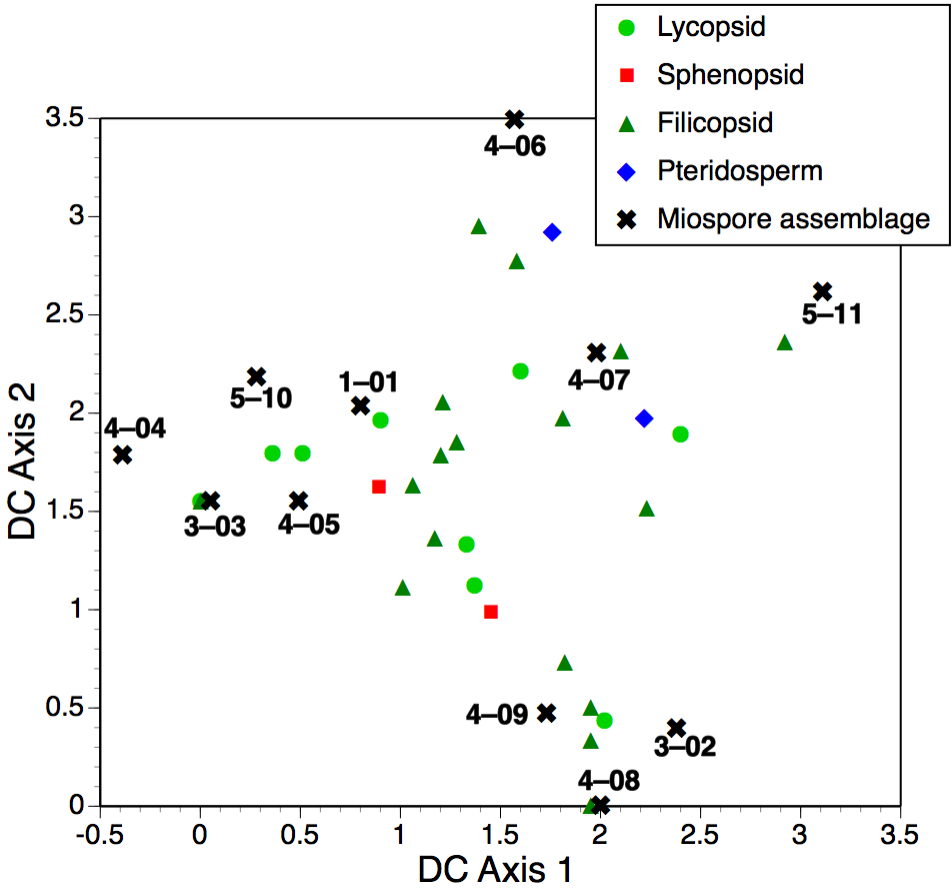
Ordination of miospore assemblages. Detrended correspondence analysis of palynological samples (crosses with black numbers indicating the source unit before the dash and the sample number after the dash) and spore genera (coloured symbols) found at Loch Humphrey Burn. Eigenvalues: Axis 1 = 0.455, Axis 2 = 0.155.

**Figure 12:**
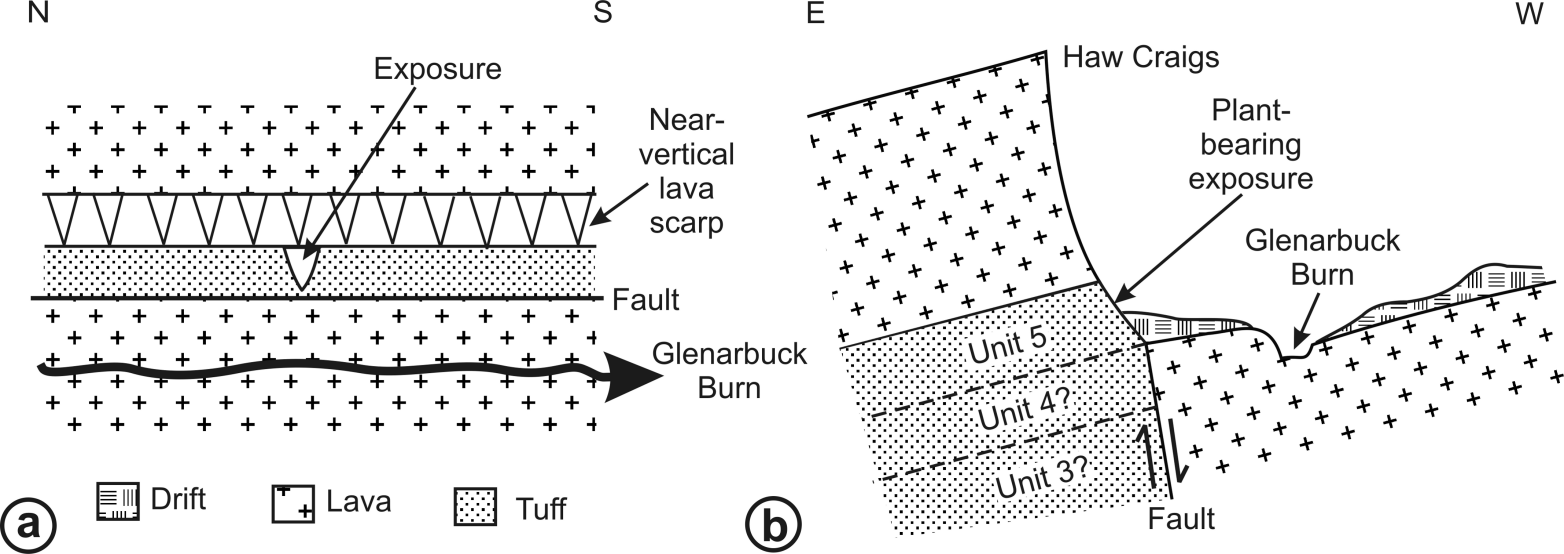
Stylised sketches of the Glenarbuck plant-bearing locality. Figures show a plan view (A) and a tentative re-interpretation of the stratigraphy (B) prompted by the re-evaluation of Loch Humphrey Burn.

**Figure 13:**
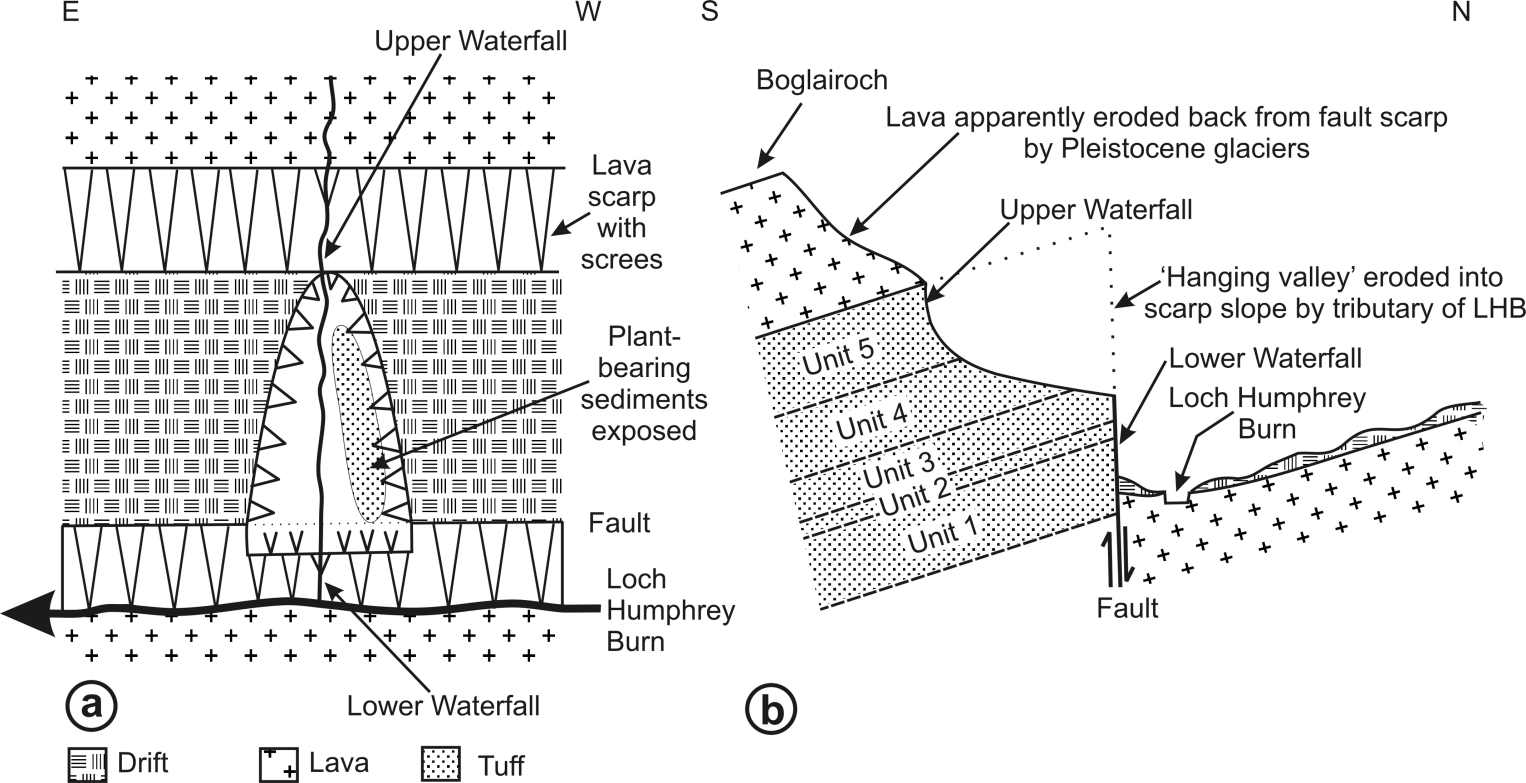
Stylised sketches of the Loch Humphrey Burn plant-bearing locality. Figures show a plan view (A) and the revised stratigraphy (B).

**Figure 14:**
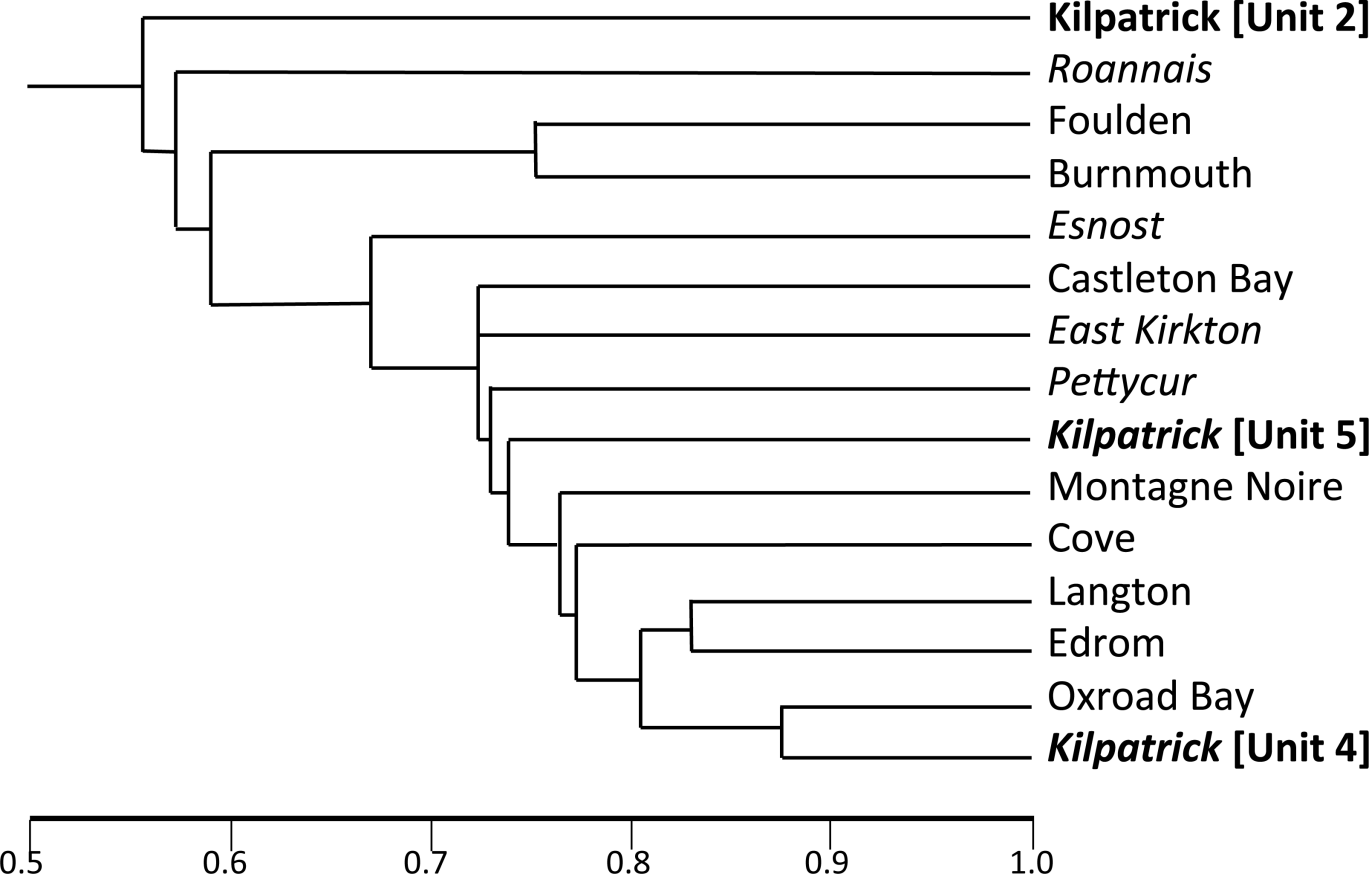
Comparative dendrogram. Bray-Curtis dissimilarity dendrogram of the spectra of putative conceptual whole-plant species reported from Mississippian lagerstätten in the Scottish Midland Valley, the Tweed Sub-basin of the Scottish Borders, and the Massif Central of south-central France. Supposedly Visean localities are italicised, unlike supposedly Tournaisian localities. The three macrofloras from the Kilpatrick Hills are highlighted in boldface.

**(a) Glenarbuck**
(i) Permineralisations (* includes some compressions)
Lycopsida: Isoetales
*Paralycopodites brevifolius* (Williamson) Morey & Morey [2] F: Smith (1962c; Text-Fig. 5, Plate 5 Figs 15–18)
*‘Lepidodendron’ solenofolium* D.L. Smith [m/S] F: Smith (1962c; Text-Fig. 4, Plate 2 Figs. 13–14)
*Lepidophloios kilpatrickensis D.L. Smith [m/S] F: Smith (1962c; Text-Figs. 1-3, Plates 1–2 Figs. 1–12)
*Lepidocarpon wildianum* Scott [1] M: Smith (1964)
cf. *Mittagia seminiformis* Lignier [?] M: Smith (1964)
*Stigmaria ficoides (Sternberg) Brongniart [m] M: Young (1873); F: Scott, Galtier & Clayton (1984; Fig. 20A)
Sphenopsida: Equisetales
*Protocalamites göppertii* (Solms-Laubach) R. M. Bateman [?] M: Smith (1964); F: Scott, Galtier & Clayton (1984; Fig. 7F)
Filicopsida: Zygopteridales
*Metadineuron ellipticum* (Kidston) Galtier [2] M: Smith (1964); F: Scott, Galtier & Clayton (1984; Fig. 7D)
*Metaclepsydropsis duplex* Williamson [?] M: Smith (1964); F: Scott, Galtier & Clayton (1984; Fig. 7D)
: Coenopteridales
*Botryopteris antiqua* Kidston [?] M: Walton, Weir & Leitch (1938); F: Scott, Galtier & Clayton (1984; Fig. 7B)
Gymnospermopsida: Pteridospermales; Lyginopteridaceae
*Heterangium grievii* Williamson [1] M: Smith (1964); F: Scott, Galtier & Clayton (1984; Fig. 7E)
*Lyginorachis brownii* Calder [1/S] F: Calder (1935; Plate 1 Figs. 8–9, 13–15)
Gymnospermopsida: Incertae Sedis
*Endoxylon zonatum* (Kidston) Scott [1] F: Lacey (1953; Text-Figs. 5–6, Plate 34 Figs. 15–16)
**(ii) Additional compressions**
Lycopsida: Isoetales
‘Lepidodendrid twigs’ [?] M: Smith (1960)
?Gymnospermopsida
*Aneimites acadica* Dawson [?] M: Walton, Weir & Leitch (1938), Smith (1960)
**(b) Loch Humphrey Burn (Units 1–2): Permineralisations**
**(i) Exposure A2 (‘Bed 1a’ of Scott, Galtier & Clayton, 1984; see ‘Unit 1: Green-grey ashy conglomerates’)**
Gymnospermopsida: Pteridospermales; Calamopityaceae
*Calamopitys radiata* Scott [1/S] F: Scott (1924; Text-Fig. 1, Plates 1–2 Figs. 1–11)
**(ii) Exposure B (‘Bed 6’ of Scott, Galtier & Clayton, 1984)**
Sphenopsida: Equisetales
*Protocalamites göppertii* (Solms-Laubach) R. M. Bateman [m] F: Scott, Galtier & Clayton (1985; Plate 1 Fig. 3, Plate 2 Fig. 2)
*Protocalamostachys arranensis* Walton (= ‘Fructification-type A’) [?] F: Scott, Galtier & Clayton (1985; Plate 3 Fig. 1, Plate 4 Fig. 1) (see Bateman, 1991; Bateman & Cleal, 1995a)
*P*. cf. *farringtonii* RM Bateman (= ‘Fructification-type B’) [?] F: Scott, Galtier & Clayton (1985; Plate 3 Figs 2–3, Plate 4 Figs. 2–3) (see Bateman, 1991; Bateman & Cleal, 1995a)
Filicopsida: Cladoxylales
*Cladoxylon* cf. *taeniatum* Bertrand [?] F: Scott, Galtier & Clayton (1985; Plate 2 Fig. 6)
*Hierogramma* sp. [?] M: Scott, Galtier & Clayton (1985)
*Syncardia* sp. [?] M: Scott, Galtier & Clayton (1985)
: Zygopteridales
*Clepsydropsis* sp. [?] F: Scott, Galtier & Clayton (1985; Plate 2 Fig. 4)
‘Fructification-type C’ (cf. *Seftenbergia* sp.) [?] F: Scott, Galtier & Clayton (1985; Plate 4 Fig. 6)
*Metaclepsydropsis* sp. [?] F: Scott, Galtier & Clayton (1985; Plate 2 Fig. 4)
‘Fructification-type D’ (cf. *Musatea* sp.) [?] F: Scott, Galtier & Clayton (1985; Plate 4 Fig. 7)
‘Fructification-type E’ (cf. *Corynepteris*) [?] F: Scott, Galtier & Clayton (1985; Plate 5 Figs. 1–2)
: Coenopteridales
*Botryopteris* cf. *antiqua* Kidston [?] F: Scott, Galtier & Clayton (1985; Plate 2 Fig. 1)
‘Fructification-type F’ [?] F: Scott, Galtier & Clayton (1985; Plate 3 Fig. 6, Plate 5 Figs. 3–5)
: ?Marattiales
*Burnitheca pusilla*Meyer-Berthaud & Galtier (1986) [?]
: Incertae Sedis
‘Fructification-type G’ [?] F: Scott, Galtier & Clayton (1985; Plate 3 Figs. 8–9, Plate 5 Figs. 6–7)
‘Fructification-type H’ [?] F: Scott, Galtier & Clayton (1985; Plate 5, Fig. 8)
?Filicopsida or Gymnospermopsida
‘Fructification-type I’ [?] F: Scott, Galtier & Clayton (1985; Plate 3 Fig. 7)
Gymnospermopsida: Pteridospermales; Lyginopteridaceae
cf. *Lyginorachis* sp. [?] M: Scott, Galtier & Clayton (1985)
: ; Calamopityaceae
*Kalymma* cf. *tuediana* Long [?] F: Scott, Galtier & Clayton (1985; Plate 2 Fig. 7)
: ; Incertae Sedis
cf. *Amyelon* sp. [?] F: Scott, Galtier & Clayton (1985; Plate 2 Fig. 3)
**(c) Loch Humphrey Burn (Unit 4): Permineralisations**
**Exposure A1 (‘Bed 17’ of Scott, Galtier & Clayton, 1984); asterisk**=**questionably assigned to this exposure**
Sphenopsida: Equisetales
*Protocalamites göppertii* (Solms-Laubach) R. M. Bateman [m] F: Walton (1949b; Text-Fig. 2, Plate 2 Figs. 11–21)
*Protocalamostachys arranensis* Walton [1] F: Bateman (1991; Text-Figs. 13C–13D, Plate 8 Figs. 72–82)
Filicopsida: Zygopteridales
*Etapteris tubicaulis* Göppert [1] M: Walton, Weir & Leitch (1938); F: Scott, Galtier & Clayton (1984; Fig. 9C)
Progymnospermopsida: Protopityales
*Protopitys scotica* Walton [3] F: Walton (1957; Plates 1–3 Figs. 1–26); F: Smith (1962b; Text-Fig. 1, Plate 34 Figs. 1–5, Plate 35 Figs. 1–3)
Gymnospermopsida: Pteridospermales; Lyginopteridaceae
*Eristophyton waltonii* Lacey [1/S] F: Lacey (1953; Text-Figs. 1–4, Plate 34 Figs. 1–4)
*Lyginorachis trinervis* Calder [1/S] F: Calder (1935; Plate 2 Figs. 16–24)
*L*. cf. *waltonii* Calder [1] M: Bateman & Cleal (1995a)
*L*. cf. *kingswoodense* Meyer-Berthaud [1] M: Bateman & Cleal (1995a, as “*L*. ?sp. nov.”)
cf. *Pullaritheca* sp. [1] M: Bateman & Cleal (1995a)
: ; ?Lyginopteridaceae
*Calathospermum scoticum* Walton [m/G] F: Walton (1940; Fig. 110; 1949a; Text-Figs. 1–3, Plates 1–3 Figs. 1–30)
*Geminitheca scotica* D.L. Smith [m/G] F: Smith (1959; Text-Figs. 1–5, Plates 1–2 Figs. 1–16)
: ; ?Calamopityaceae
*Kalymma* sp. [1] M: Bateman & Cleal (1995a)
*Alcicornopteris hallei* Walton [2/S] F: Walton (1949c; Plates 12–13 Figs. 1–13)
**(d) Loch Humphrey Burn (Units 4–5): Compressions**
**Exposure A1 (‘Beds 13–33’ of Scott, Galtier & Clayton, 1984)**
Lycopsida: Isoetales
*Lepidophloios* cf. *kilpatrickensis* D.H. Smith [1] M: Bateman & Cleal (1995a)
*Stigmaria ficoides* (Sternberg) Brongniart [?] M: Smith (1964)
Sphenopsida: Equisetales
*Archaeocalamites radiatus* (Brongniart) Stur [m] F: Walton (1949b)
*Pothocites grantoni* Paterson [?] M: Smith (1964); F: Chaphekar (1965)
?Filicopsida: Incertae sedis
*Rhodeopteridium* sp. Zimmermann [?] M: Walton (1957)
Gymnospermopsida: Pteridospermales: ?Lyginopteridaceae
*Rhacopteris lindsaeformis* Bunbury [m] ?F: Kidston (1923–5)
*R. inaequilatera* Göppert [m] ?F: Kidston (1923–5)
*R. robusta* Kidston [?] M: Walton, Weir & Leitch (1938)
*R. petiolata* Göppert [?] M: Walton (1959)
*Sphenopteridium pachyrrachis* (Göppert) Schimper [m] ?F: Kidston (1923–5)
*S. crassum* (Lindley & Hutton) Kidston [?] ?F: Kidston (1923–5)
*Sphenopteris affinis* Lindley & Hutton [?] ?F: Kidston (1923–5)
*S. bifida* Lindley & Hutton [?] ?F: Kidston (1923–5)
*Calathiops trisperma* D.L. Smith [2/S] F: Kidston (1923–5)–5) [as *Pterispermatostrobus* sp.]; F: Smith (1962b)
: ; ?Calamopityaceae
*Spathulopteris ettingshausenii* (Feistmantel) Kidston [?] ?F: Kidston (1923–5)
*S. obovata* (Lindley & Hutton) Kidston [?] ?F: Kidston (1923–5)
*Staphylotheca kilpatrickensis* D.L. Smith [1/G] F: Smith (1962b)
*Alcicornopteris zeilleri* Kidston [?] ?F: Kidston (1923–5)
*A. convoluta* Kidston [?] F: Walton (1949c; Plate 12 Fig. 1)
**(e) Loch Humphrey Burn (Unit unknown): Permineralisations**
Gymnospermopsida: Pteridospermales: ?Lyginopteridaceae
*Eristophyton fasciculare (Scott) Zalessky [1/S] M: Scott (1899); F: Scott (1902; Plate 1 Figs. 1–7, Plate 3 Figs. 1–5, Plate 4 Figs. 6–7); F: Scott (1918; Plate 8 Fig. 23)
*Bilignea resinosa Kidston [1/S] M: Scott (1923); F: Scott (1924; Plates 5–6, Figs. 36–40)

As at Glenarbuck, following a hiatus, palaeobotanical interest in Loch Humphrey Burn was rekindled by Robert Brown, Jessie Watson and John Walton between 1930 and 1935 (Liston & Sanders, 2005). The permineralisations, partial permineralisations and compressions that they acquired were subjected to palaeobotanical study throughout the mid-20th Century by Calder (1935), Walton, Weir & Leitch (1938), Walton (1940), Walton (1949b), Walton (1957), Lacey (1953), Smith (1959), Smith (1960), Smith (1962a), Smith (1962b), Smith (1964), Chaphekar (1963) and Chaphekar (1965), resulting in the description of seven types, three of which were generitypes. The assemblage was dominated by several pteridosperms, together with a probable progymnosperm (*Protopitys scotica*), a sphenopsid (*Protocalamites goeppertii*) and a single fragment of a fern phyllophore (*Etapteris tubicaulis*) (Tables 1D and 1E). All of these specimens were probably found in “a small but rich … lenticular bed of fine sandstone … in shales … a few feet above the bed of volcanic ash containing *Bilignea resinosa* and *Calamopitys radiata*” (two of the three species of pteridosperm stem described by Scott: see Walton, 1949b; Walton, 1949c), by “a small stream on the south side of Loch Humphrey Burn” (Lacey, 1953) and “above a small waterfall” (Walton, 1949a).

In 1979, Exposures A1 and A2 were trenched and logged by a team led by Andrew Scott. They also trenched a new exposure, here termed Exposure B, 60 m east of Exposure A. This revealed several layers of pteridosperm-dominated compressions and an associated horizon of small calcareous nodules containing permineralisations (‘Bed 17’ of Scott, Galtier & Clayton, 1984) that resembled those obtained from Exposure A1 by Brown and Walton. An additional horizon of calcareous nodules containing permineralisations (their ‘Bed 6’) was found 7 m below ‘Bed 17’ (Scott, Galtier & Clayton, 1984; Scott, Galtier & Clayton, 1985). Plants identified in ‘Bed 6’ included *Protocalamites*, several fern taxa (some of these are earliest known occurrences in the fossil record, sporangia being particularly well represented: Meyer-Berthaud & Galtier, 1986) and foliage of both calamopitid and lyginopterid pteridosperms (Scott, Galtier & Clayton, 1985; Scott & Galtier, 1996) (Table 1B).

Miospore dating showed that the upper flora at Site B is mid-Visean (Scott, Galtier & Clayton, 1984) and assignable to the TS palynozone (Clayton, 1985); that is, latest Arundian–Holkerian stages (Fig. 2). Permineralised plants of this age have not been recorded in Britain outside the Kilpatrick Hills (Scott, Galtier & Clayton, 1984). Surprisingly, the lower flora at site B was documented as representing the late Tournaisian (Ivorian = late Courceyan Stage, Tn3) CM palynozone (Fig. 2), indicating that it was penecontemporaneous with several better-known floras that were located in East Lothian and Berwickshire, around the southeastern margin of the Scottish Midland Valley (Scott, Galtier & Clayton, 1984; Scott, Galtier & Clayton, 1985; Scott, 1990; Scott & Galtier, 1996). Consequently, the lack of knowledge concerning the precise origin of the three stem genera described by Scott (1899, *et seq.*) means that their age is uncertain (Scott, Galtier & Clayton, 1984). Scott (1924) himself was evidently confused, since he attributed *Calamopitys radiata* and *Bilignea resinosa* to the same bed but ascribed the former to the “Oil Shale Series” and the latter to the older “Calciferous Sandstone Series” (Smith, 1960).

In the first (albeit brief) account of the local palaeoecology, Smith (1964) speculated that the pteridosperm-dominated upper Loch Humphrey Burn flora represents a back-swamp forest community, drier than the lycopsid-dominated putative swamp flora documented nearby at Glenarbuck. In contrast, Scott, Galtier & Clayton (1985) envisaged the lower Loch Humphrey Burn flora of their ‘Bed 6’ as a pioneer community occupying a virgin volcanigenic terrain.

## Re-survey and Re-excavation of Loch Humphrey Burn

During April–May 1985, RMB crudely surveyed all the areas of volcanic ash in the Kilpatrick Hills indicated on the 6” field slips used by Jack (1870–2) to prepare the 1” Geological Survey map of Glasgow (Sheet 30). All sedimentary exposures encountered were then briefly searched for plant remains, and any promising exposures were sampled for subsequent laboratory examination.

Both of the ‘horst-block inliers’ indicated in Fig. 3 yielded several sites for poorly preserved compressions; most were stigmarian rootlets of arborescent lycopsids, apparently *in situ* and marking palaeosols (e.g., NS439757, 440756, 453760, 459756, 463755). However, the more interesting exposures occurred along the westernmost lava scarp, Lang Craigs (e.g., NS430760, *ca* +180 m asl; 431764, *ca* +210 m asl), where buff-coloured siltstones and fine sands with thin impersistent, organic-rich shales (‘coals’) crop out immediately below the lavas (admittedly, they are largely obscured below by screes). These sediments include several horizons of calcareous concretions that vary considerably in size and degree of induration. Examination of numerous concretions revealed ubiquitous permineralised stigmarian rootlets, two weakly permineralised *Stigmaria* cf. *ficoides* root branches and a single fragment of a probable *Lepidocarpon* strobilus. These assemblages most likely represent contemporary wetland soils.

However, plant assemblages from these localities were judged insufficiently diverse and/or well preserved to merit detailed study. Although the classic localities at Loch Humphrey Burn and, especially, Glenarbuck had become severely weathered and largely obscured by talus, RMB decided to concentrate a substantial part of his doctoral research (Bateman, 1988; see also Bateman, 1991; Bateman & Cleal, 1995a; Bateman & Cleal, 1995b) on Loch Humphrey Burn, for the following reasons:

(1) Its diverse recorded flora of both compressions and permineralisations (Table 1) includes several species that are unique to the locality and have figured prominently in discussions on early seed-plant evolution (e.g., *Protopitys scotica*, *Calathospermum scoticum*, *Germinitheca scotica*).
(2) At least one plant-rich horizon demonstrably contained compressions and permineralisations preserved in intimate association, apparently presenting an exceptional opportunity to prove attachment of compressed and permineralised organs and thus at least partially reconstruct conceptual whole-plants (cf. Chaloner, 1986; Galtier, 1986; Bateman & Rothwell, 1990; Bateman & Hilton, 2009).
(3) Although this opportunity had long existed, physical connection had previously been demonstrated between only two pairs of small organs: the permineralised sporangia and partially compressed sporangiophore of *Alcicornopteris hallei*–*A. convoluta* (Walton, 1949b), and the partially permineralised rachis and compressed sporangia of the progymnosperm *Protopitys scotica* (Walton, 1957).
(4) As previous palaeobotanical studies were based on a relatively small amount of material collected before 1935, it seemed probable that further collecting, preceded by re-excavation and followed by the application of modern analytical techniques, would not only provide new information on species known to occur at the locality but would also reveal species new to the Kilpatrick Hills (or even new to science).
(5) A miospore profile spanning 30 m of sediment (Scott, Galtier & Clayton, 1984) had recently become available, potentially allowing high-resolution relative dating of individual beds (and therefore of plant-bearing horizons). This in turn suggested that Loch Humphrey Burn and Glenarbuck contain the only known mid-Visean permineralised floras in Europe, and are exceptional in reputedly spanning the contentious Tournaisian–Visean stage boundary.

Preliminary inspection of the classic Loch Humphrey Burn locality (Exposure A1) showed that a much more ambitious restoration programme was needed to elucidate its stratigraphy, identify the horizons collected by previous workers and enable collection of new material. This work was funded by the then UK Nature Conservancy Council; it was performed in September–October 1985 by RMB and two paid assistants, Robert Gray and Gordon Barrass:

(1) A face 26 m long and up to 18 m high (Exposure A1) was manually cleared of talus and photographed (Figs. 4 and 5).
(2) Selected profiles cut back to unweathered rock provided three lithological logs (Profiles 2–4, Fig. 6) that recorded texture, lamination intervals, degree of induration, colour (as per the Munsell Corporation chart), and plant species present (these lists were later expanded during preliminary laboratory inspection of the materials collected). A fourth log (Profile 1, Fig. 6) was made of the underlying strata at Exposure A2 (Fig. 4C). Measured thicknesses of these beds were subsequently reduced in Fig. 7 to compensate for dip away from the observer.
(3) Promising plant-bearing horizons identified during (2) were further investigated, and preliminary collections were taken from nine levels (Floras 1–9, Figs. 6 and 7). Approximately 250 kg of fossiliferous sediment was subsequently collected from the most promising Floras, 1 and 4. Permineralisation-bearing nodules were extracted later in the laboratory (Fig. 8; see ‘Synthesis of newly-extracted permineralised plants’), before being placed in long-term storage at the National Museums of Scotland, Edinburgh.

The most recent re-excavation of portions of the Loch Humphrey Burn site was conducted by all three of the present authors in September 2006. Given that considerable thicknesses of talus had accumulated during the 21 years that had elapsed since the major excavation by RMB in 1985, the team decided to focus on re-exposing the three sections that had been logged in 1985 at Exposure A1 (Fig. 5B). The primary objective was to sample each horizon for subsequent palynological investigations by LGS, though the opportunity was also taken to remove a modest number of additional permineralisation-bearing blocks.

## General Site Description

Figure 4C shows the relative positions of the three Exposures, A1, A2 and B. Profile 1 (Fig. 6) shows the lithology of the waterfall section, Exposure A2 (Fig. 4C). Logging of the waterfall section largely followed Scott, Galtier & Clayton (1984, Fig. 8), but was revised following the partial re-excavation in 2006. Exposure A1 (Figs. 4C, 4D and 5) runs perpendicular to, and is stratigraphically higher than, Exposure A2. These two exposures were sufficiently close to allow tracing of the relatively distinctive ‘Fish Bed’ from the top of Exposure A2 to the base of Exposure A1. Figure 5B shows the most prominent lithological boundaries and structures in Exposure A1. The beds dip more-or-less southwards at an angle that gradually decreases from *ca* 20° for the lowermost beds to *ca* 10°for the uppermost. Several minor faults, typically with downthrows of *ca* 30 cm, occur approximately perpendicular to both the bedding planes and the face of the exposure, and other less obvious fault planes run approximately parallel to the face. Both sets of faults are mirrored by abundant minor fractures throughout the sediments. Mississippian sediments at the top of the exposure are thinly blanketed with Pleistocene subglacial till, and are severely weathered to a depth of *ca* 2 m.

Three profiles were recorded from Exposure A1 (Figs. 5B and 6). Profiles 2 and 4, at the extremities of the exposure and hence *ca* 26 m apart, show only slight stratigraphic overlap, but both profiles overlap considerably with an intermediate transect (Profile 3) that was logged to assess the degree of lateral variation in the various stratigraphic units. Profiles 2–4 can be compared with the lithological log for Exposure B published by Scott, Galtier & Clayton (1984: their ‘Beds 6–33’). Sediments stratigraphically higher than the top of Profile 4 were not examined in detail, but their contact with the overlying basaltic lavas was excavated and the thickness of the sediment between the top of Profile 4 and the base of the lavas was estimated later, from photographs. The total thickness of sediments described, from the base of the waterfall to the base of the lavas, is 30 m.

The detailed lithological logs presented in Fig. 6 are summarised in a more generalised composite log (Fig. 7). This shows that the sediments can usefully be divided into five main lithological units (Units 1–5), which are described below in order of decreasing age. Information given in parentheses for each unit is (a) the identity of the exposure that it encompasses, (b) its palynologically estimated age, (c) its observed maximum thickness, and (d) suggested correlation with the numbered beds at exposure B logged by Scott, Galtier & Clayton (1984, Fig. 8). As the discussions on ‘Provenance and depositional environments’ for each depositional unit on pages 21–26 are based largely on field observations, validation via detailed laboratory analyses remains desirable. The five units together encompassed nine beds bearing identifiable plant fossils (Floras 1–9: Fig. 7).

## Unit 1: Green-grey Ashy Conglomerates

(A2; late Tournaisian; 12 m; ‘Beds 1–5’ (logged at site A2))

### Lithology

Unit 1 contains a series of poorly laminated, poorly sorted, coarse, greenish-grey sandstones, rich in volcanic ash and often containing large (≤20 cm) rounded lava clasts; these are interrupted by only one thin shale.

### Plant remains

A 60 cm-thick horizon near the base (within Bed 1a of Scott, Galtier & Clayton, 1984) contains abundant fragments of gymnospermous wood (Flora 1), some fragments evidently derived from large trees; these were overlooked by AC Scott et al. Although permineralised, these woods are poorly preserved, having suffered extensive calcite recrystallisation and pyritisation. The lithology of this bed is consistent with the “coarse ash agglomerate” that reputedly provided Scott (1899, *et seq.*) with his three new species of woody stem; the poor quality of preservation of his *Calamopitys radiata* in particular strongly resembles that of stems extracted from the bed in 1985 by RMB (Figs. 8A and 8B).

### Provenance and depositional environments

The abundant volcanic ash and lava cobbles, progressively more weathered when traced upward, suggest that these sediments were eroded from a nearby volcanic terrain. The poor lamination and sorting, presence of both large clasts and at least one erosional contact, and the random orientation of the wood fragments within the horizontal plane all indicate high-energy fluvial transport and rapid, chaotic deposition. The fragmentation, rounding and poor preservation of the woods together suggest that they were transported over a considerable distance.

## Unit 2: Brown Inorganic Siltstones and Fine Sands

(A1 + B; late Tournaisian∼early Visean; 2 m; ‘Beds 6–11’)

### Lithology

This relatively thin unit consists of largely unfossiliferous, putatively late Tournaisian laminated siltstones and fine sands overlain by the putatively early Visean secondary limestone and dark grey argillaceous ‘Fish Bed’ of Scott, Galtier & Clayton (1984: ‘Bed 11’), which they reported as yielding fish scales, arthropod cuticle and ostracods. The lowest bed in Unit 2 (‘Bed 6’ of Scott, Galtier & Clayton, 1984) contains ≤15 cm calcareous nodules surrounded by distinctive purple clay rinds, and crops out at both Exposure A1 (Figs. 4 and 6) and Exposure B.

### Plant remains

The calcareous nodules found in ‘Bed 6’ at Exposure B and in the equivalent bed at the base of Exposure A1 (Flora 2) contained small permineralised plant fragments. These were poorly preserved and unidentifiable at Exposure A1 but at Exposure B they yielded a diverse fern–sphenopsid–pteridosperm flora (Scott, Galtier & Clayton, 1985).

### Provenance and depositional environment

These finer, prominently laminated sediments appear to have been deposited in a lower energy flow regime than the underlying conglomerates, and are poorer in recognisable volcanigenic clasts. The lower siltstones are probably dominantly alluvial flood-plain deposits, though the overlying secondary limestone and dark shale rich in animal fossils were interpreted as lacustrine by Scott, Galtier & Clayton (1984). The small, permineralised plant fragments are frequently burnt, clearly detrital, and could therefore have been transported over considerable distances (Scott, Galtier & Clayton, 1985).

## Unit 3: Dark Grey Shales and Siltstones

(A1 + B; (early or) mid-Visean; 3.65 m; ‘Bed 12’)

### Lithology

Unit 3 consists of comparatively uniform, thinly laminated, dark grey silty shales. In the central part of the unit 1.25 m of sediment has formed ‘pseudo-nodules’: these large (*ca* 30 cm), flattened, brick-like structures consisting of thinly laminated silty shales are surrounded by several concentric layers of similar sediment but lack appreciable cementation. A similar but much thinner horizon occurs near the top of the unit (Fig. 7).

### Plant remains

Unit 3 contains abundant compressed plant remains throughout; they are preserved as dark grey, oily organic ‘smears’ that do not markedly contrast with the matrix and are therefore comparatively difficult to identify taxonomically. The entire unit is here treated as a single assemblage (Flora 3). It is dominated by many of the species of pteridosperm foliage and reproductive organs that characterise Unit 4, though RMB also exhumed a single specimen of the arborescent lycopsid rootstock *Stigmaria ficoides*. The plant remains are generally more complete toward the top of the unit, where compressed portions of fern-like fronds, petioles and stems are particularly frequent.

### Provenance and depositional environment

Unit 3 resembles Unit 2 in texture and lack of volcanic clasts, and is possibly also an alluvial flood-plain deposit. However, it has a much greater organic content that includes large, delicate plant fragments, suggesting that this was fed by a richer, and hence presumably more proximal, source of plant material.

## Unit 4: Green Ashy Sandstones

(A1 + B; mid-Visean, 4.75–5.75 m; ‘Beds 13–20’)

### Lithology

Unit 4 exhibits considerable lateral and vertical heterogeneity (Figs. 5 and 6), though its basic stratigraphy can be traced across Exposure A1 and thereby correlated tentatively with the succession at Exposure B. Both its uppermost and lowermost beds are coarse, gritty sandstones rich in volcanic ash. The lowermost bed fines upward from a basal conglomerate; at Exposure A1 this appeared to overlie Unit 3 conformably in Profiles 3 and 4 but unconformably in Profile 2. These coarse sandstones are separated by several beds of medium to coarse sandstone (the upper beds ashy in Profiles 3 and 4) intercalated with between three and five thin (≤5 cm) brownish-grey, mildly indurated shales that are best developed in Profile 3 (Fig. 6). The sandstones show only slight induration in Profile 2, but Profile 3 contains two thin concretionary horizons, and half the total thickness of Unit 4 is strongly cemented in Profile 3, including the 1.5 m thickness immediately above its base. This induration is reflected in a 1 m increase in the overall thickness of the unit between Profiles 3 and 4, so that the southward dip of the unit is greater at its base than at its top (Fig. 6). In Profile 4, the lower part of the unit also includes two horizons of sparse, small nodules: the first of calcium carbonate, 5 cm above the basal conglomerate and the second of chert, 1.3 m above the basal conglomerate (Fig. 6). Both the concretionary layers and the nodules rapidly peter out northward.

### Plant remains

Unit 4 is the most palaeobotanically significant portion of the Kilpatrick Hills sequence; most of the constituent beds of this unit contain plant remains. Several beds, particularly in the upper part of the unit, yielded only fusainised fragments, but five contained readily identifiable material (Floras 4–8, Fig. 7); four of these occurred within, and immediately above, the thin brownish-grey shales. In addition, the gritty sandstones at the top and bottom of the unit contained several three-dimensional infilled stems of *Protocalamites*, some of which were partially permineralised and reached 2 cm in diameter.

The sediments containing Flora 4 show considerable lateral variation. In the southern-most profile (4) they consist of 3 cm of brownish-grey shale, coarsening up into 7 cm of fine–medium sand (Fig. 4D) that is blue-grey when fresh but oxidises to green within a few days of exposure. This is the internationally important “lenticular bed of fine sandstone” that was extensively collected by Walton and co-workers in the mid-20th century and yielded most of the famous permineralisations and compressions recorded at Loch Humphrey Burn; the flora is dominated by several pteridosperms but also contains at least one progymnosperm, one fern and one sphenopsid (Table 1D). The high-quality permineralisations occur within the fine sands as a single horizon of small (the longest dimension does not exceed 8 cm with a median of 3–4 cm) calcareous nodules that appear to have nucleated around plant material (e.g., petiole fragments, cupules: Fig. 8). The nodules were associated (often intimately) with abundant well-preserved compressions—some subtended by partially permineralised axes—and occurred at the extreme southern end of the exposure. Unfortunately, the nodules terminated at a small fault located only 1 m north of the southern end of the exposure. Moreover, the most accessible parts of this bed had previously been extracted early in the 20th century, leaving a near-vertical face within which Flora 4 immediately underlies 1.5 m of strongly cemented sandstones. Extraction of further plant-bearing material was consequently extremely laborious, so it was possible to collect only a limited number of fragmented blocks of this highly desirable fossiliferous material.

The shale at the base of Flora 4 could be traced reliably across the exposure (except for a small section between Profiles 2 and 3) to the northern extremity, but the distinctness and richness of the associated uncemented fine sands decreased rapidly northward. This lateral change in lithology was accompanied by decreased quality and increased weathering of the compressions; both are probable consequences of the petering out of the overlying concretionary horizons (Figs. 6 and 7).

Flora 5 consisted of sporadic dark brown to black compressed plant fragments, mostly fern-like foliage, dispersed among several centimetres of green, medium-grained sandstones 1–1.5 m above Flora 4. These were cemented in Profile 4 but uncemented further north.

Flora 6 occurred *ca* 2.3 m above Flora 4, in 2–3 cm of brownish-grey indurated shale located immediately below several centimetres of greyish-green medium sand. This horizon could be traced for *ca* 16 m from the south end of the exposure (where it was sandwiched between cemented horizons: Fig. 6B), but only the central section was plant-rich (Profile 3; Figs. 6 and 7). Its diverse compression flora resembled that of Flora 4 in species composition (Table 1E), preservation and degree of fragmentation.

Floras 7 and 8 occurred in sediments similar to, and deposited immediately above, Flora 6. They were located in the central part of the section, 10 cm and 55 cm respectively above Flora 6 (Fig. 7). The only recognisable species found in either flora was the compressed, ‘fern-like’ foliage *Rhacopteris lindsaeformis*.

Unfortunately, beds and floras of Unit 4 are difficult to correlate between Exposures A1 and B. At Exposure B, a horizon within ‘Bed 17’ of Scott, Galtier & Clayton (1984) yielded both compressions and permineralisations, and was lithologically similar to the horizon containing Flora 4 at Exposure A1; 2 cm of brownish-grey shale underlay several centimetres of green medium-grained sand that enclosed a horizon containing small calcareous nodules. It also yielded most of the compressed and permineralised organ-species found in Flora 4. However, a similar compression flora has been recorded at a stratigraphically higher position in Exposure A1 (Flora 6: Table 1E), and the central stratigraphical position of ‘Bed 17’ at Exposure B appears to resemble more closely that of Flora 6 than Flora 4 at Exposure A1. Thus, Scott et al.’s ‘Bed 17’ may correlate with Flora 4, or with Flora 6, or even potentially with neither of the more recently (re-)exposed floras.

### Provenance and depositional environments

Unit 4 clearly represents a return to deposition of coarse, hydraulically reworked volcanigenic material, although it lacks the frequent lava cobbles observed in Unit 1. These sediments appear to have been deposited in a high-energy flow regime; Scott, Galtier & Clayton (1984) interpreted them as channel deposits. On the basis of our descriptions, WA DiMichele (pers. comm., 2015) suggested that the small scale of the channels and infra-formational location of the reworked cobbles together suggest a ‘flashy discharge’ regime. The most desirable plant remains occur in thin intercalated beds of finer sediment that presumably represent periods of lower energy flow and slower accumulation of sediments. The best-preserved and most intact plant remains, which include large frond segments and cupule clusters, occur in thin fine–medium sand horizons above thin shales and below thicker, coarser sandstones (i.e., within coarsening-up sequences). This suggests that they accumulated early in a phase of increasing flow and were deposited close to their source, before the plant remains could suffer appreciable fragmentation and decay; they were then buried rapidly by increasingly coarse sandstones. Rapid cementation, primarily with calcium carbonate, occurred in the southern part of the exposure; the highly localised nature of the cementation is inexplicable on present evidence and merits more detailed study (see ‘Synthesis of newly-extracted permineralised plants’).

## Unit 5: Brown Organic Siltstones and Fine Sands

(Site A1 + B; mid-Visean; *ca* 12 m; ‘Beds 21–33’)

### Lithology

Only the lowermost 7 m of this unit was logged in detail, mainly in Profile 4 (Figs. 6 and 7). In this profile, the lowermost 4 m are lithologically transitional from Unit 4; the greenish-grey and greenish-brown siltstones and fine sand that characterise Unit 5 are intercalated with three 18–32 cm-thick, largely ash-free, medium–coarse sandstones (two of them cemented) and one 64 cm-thick ash-rich medium sandstone. A 60 cm-thick siltstone bed immediately above this sandstone contains three thin (≤5 cm) organic shales that can be traced laterally until obscured by drift deposits north of Profile 3 (Figs. 5 and 6). Several thin brown shales with incorporated fusainised plant fragments and thin impersistent organic-rich black shales occur between the uppermost coarse sandstone and the lavas.

### Plant remains

The lowermost bed of Unit 5, a cemented greenish-grey medium sandstone, contains Flora 9 (Fig. 7). This yielded abundant *in situ* stigmarian rootlets, plus single specimens of *Stigmaria* cf. *ficoides* and a leafy twig of a lycopsid. Unit 5 is also the probable source of a loose block found at the foot of the exposure that featured a well-preserved *Lepidophloios* axis (a small stem or, more likely, a large branch), possibly the compressed equivalent of the permineralised *L. kilpatrickensis* described by Smith (1962c) from Glenarbuck (Table 1A). Abundant plant remains occurring higher in the unit are mostly fragmented and have been severely weathered, probably during both the Mississippian (q.v. the dark organic shales) and the Holocene.

### Provenance and depositional environments

Taken together, the overall facies of Unit 5 suggest a return from the channel-reworked volcanigenic sandstones of Unit 4 to lower energy alluvial flood-plain environments. However, this transition was evidently gradual; the lowermost 4 m of Unit 5 includes four medium–coarse sandstones that resemble in lithology those of Unit 4 but enclose *in situ* stigmarian rootlets and other lycopsid remains. Poorly-developed palaeosols containing *in situ* rootlets occur throughout Unit 5, delimiting several periods of landscape stability and indicating the presence of persistent *in situ* lycopsid-dominated communities, probably occupying low-lying swamps that were subject to periodic inundation.

## Synthesis of Newly-Extracted Permineralised Plants

### Extraction and preparation of permineralised plants from Units 1, 2 and 4

The large number of blocks extracted from Unit 4 at Loch Humphrey Burn were slowly dried in the laboratory and then brushed free of surficial dirt. Subsequent cleaving along bedding planes attempted a difficult balance between the desire to expose as much plant material as possible (both compression fossils and permineralised nodules) and the fear of causing excessive fragmentation along the two inter-penetrating sets of closely-spaced joints that characterised those blocks. Further exposure of compression fossils was achieved by dégagement using mounted needles. Cleaving occasionally revealed small concretionary nodules (these had already been removed from the matrix of corresponding material previously collected from ‘Bed 17’ of exposure B by AC Scott). Initially, the nodules from exposure A1 were removed from the matrix with care, but it soon became apparent that permineralised plants within the nodules could not be correlated with compressions on the surrounding bedding plane due to diagenetic disruption of the surrounding matrix during formation of the nodule. The bulk of the collection remains uncleaved. Supposedly Tournaisian blocks taken from Unit 1 in exposure A2 were less well-bedded and more indurated than the overlying Visean material. Numerous wood fragments (some large) were excised from the matrix, which was then discarded (the few compressions present were judged to be of unacceptably poor quality).

Each nodule/wood fragment was viewed perpendicular to the bedding, its outline sketched, and its length (*c* axis) and width (*b* axis) were recorded in mm. One end was arbitrarily designated ‘Top’, the opposite end ‘Bottom’. Nodules had often nucleated around stems or branches, so an initial cut perpendicular to the *c* axis of the nodule was most likely to provide a transverse section of the target plant organ(s). Subsequent cuts with thin-bladed saws (width of cut = 1.0–1.2 mm) usually paralleled the first cut and were spaced 8–10 mm apart. Each cut surface was subjected to peeling using 50 µm-thick acetate (Galtier & Phillips, 1999) in 5% HCl for 40 ± 15 s (this etching time—fast relative to the modest amount of carbonate present in the nodule, at least for British Mississippian floras—was attributed to the nodule’s unusually high porosity by Bateman, 1988). Specimens demonstrated by these reference peels to be of particular palaeobotanical interest were embedded in Bioplastic if especially delicate. Each specimen was then subjected to serial peeling via one of two grinding regimes: either F280 carborundum followed by F800 aluminium oxide (typical spacing between successive peels was 340 ± 70 µm) or, for especially small specimens such as sporangia containing spores, F800 aluminium oxide alone (typical spacing 145 ± 25 µm).

During the course of this investigation a total of 151 calcareous nodules containing anatomically-preserved plants were collected by RMB and AC Scott, from a total of six exposures at three localities (Bateman, 1988, Tables 2.1–2.3). The 21 nodules derived from Lang Craigs generated 88 reference peels, the four nodules collected from Glenarbuck yielded 14 reference peels, and the supposed Tournaisian plant-bearing bed of Unit 1 at Loch Humphrey Burn yielded eight specimens (most subsequently discarded) and 42 reference peels. All of these sample sets were of poor preservation and low species diversity. In contrast, nodules extracted from the classic Walton Bed of Unit 4 (most likely equating with ‘Bed 17’ of Scott, Galtier & Clayton, 1984) were of medium to good preservation and much higher species diversity. A total of 118 nodules (58 collected by AC Scott) (Figs. 8D–8I) yielded 410 reference peels, and a small selection of the most promising specimens together yielded *ca* 1,000 serial peels. As yet, illustrations of only one of these specimens (of the archaeocalamitean cone *Protocalamostachys arranensis*: Fig. 8I) have been published (Bateman, 1991).

In addition, the geochemical changes associated with the permineralisation process were inferred by comparison of bulk geochemical analyses of a nodule and its surrounding matrix (see below; full technical details were provided by Bateman, 1999).

### Diagenesis of permineralised plants in Unit 4

Additional research was performed on the permineralised plants from the lower part of Unit 4, with particular reference to the permineralised archaeocalamite specimens studied by Walton (1949b) and Bateman (1991). The boundaries of these modest-sized nodules are somewhat gradational, the nodules clearly being composed largely of the same kinds of mineral grains as the enclosing grey-green, fine-sand ashes. The dominantly calcareous cementation is imperfect, generating nodules that are noticeably porous and somewhat friable; this texture could indicate at least superficial pedochemical weathering, presumably during the Quaternary.

Two small rock specimens were therefore selected by Bateman (1988) for bulk geochemical analysis: the first an indurated specimen from 1 cm depth within a permineralisation-bearing nodule and the second of unindurated sediment sampled from a point in the rock matrix 1 cm outside the same nodule. Inductively-coupled plasma-atomic emission spectrometry (ICP–AES: e.g., Gill, 1996) was used to quantify the ten widely recognised major oxides, supported by separate analyses for water content, loss-on-ignition (allowing estimation of organic carbon content) and carbonate content (Bateman, 1999) (Table 2).

**Table 2:** Oxide/element compositions. ICP–AES data for the ten common rock-forming elements (as weight % oxides) from the Walton bed (Unit 4, Flora 4) of the Visean sequence at Loch Humphrey Burn. The relatively unconsolidated reworked tuff containing compression fossils is compared with enclosed, diagenetically generated concretionary nodules containing permineralised plants. SiO_2_ is used as a notional constant in order to estimate the relative degrees of concentration of other oxides in the nodules containing anatomically preserved plants, and CO_2_ is given as CaCO_3_ equivalent by acid digestion. Also given are values (ppm) of selected minor elements totalling 0.03–0.04% of each sample. LOI, loss on ignition; NA, not applicable (see also Bateman, 1999).

Oxide	Sediment	Concretion	Change (%)
H_2_O	5.1	2.8	NA
LOI	5.5	6.1	NA
CO_2_	*ca* 0.5	28.2	NA
CaO	1.90	24.22	+1628
MgO	2.46	3.02	+66
FeO	10.52	7.25	−7
Na_2_O	0.45	1.69	+409
K_2_O	1.42	0.91	−13
P_2_O_5_	0.24	0.22	+24
MnO	0.10	0.37	+402
Al_2_O_3_	15.28	12.70	+13
TiO_2_	2.47	1.55	−15
SiO_2_	65.16	48.07	0 (fixed)
Zn	123	78	−14
Pb	<25	<25	NA
Cu	41	42	+39
Ni	83	60	−2
Cr	131	83	−14
Cd	7	4	−22
Co	29	17	−20
Mo	<1	<1	NA

The primary objective of comparing the two samples was to infer which elements most likely migrated during syn- and/or post-depositional diagenesis to form the nodules (and thus preserve the permineralised plants) and in what relative proportions. The approach used was developed by Bateman (1988) but not published until several years later (Bateman, 1995; Bateman, 1999). It has not found favour with some geochemical purists (e.g., N Trewin, pers. comm., 2006), but nonetheless experience has shown that it reliably yields credible results. The method assumes that SiO_2_, a component of many of the more diagenetically resistant minerals (most notably the sole constituent of quartz), has not migrated and thus should be a constant proportion of both the nodules and the surrounding less indurated sediment. The observed ratio of matrix SiO_2_ to nodule SiO_2_ (1.36:1) was then used to calculate the expected percentages of all other major element oxides and eight selected minor elements in the diagenetic nodules (totalling 0.03–0.04%), and the percentage divergence in the nodule from the expected value was noted (Table 2).

Any strongly negative divergences would indicate violation of the key assumption that SiO_2_ had not migrated, but in fact no negative deviations exceed 20% for the present data-set. Most of the positive deviations can similarly be discounted as falling within the presumed margin of error of this rough-and-ready method, but four oxides show significantly greater concentration in the nodules. Four-fold increases in MnO and Na_2_O, and a more modest increase in MgO, are evident, though all three elements occur at low levels relative to SiO_2_, Al_2_O_3_ and CaO. Most notably, CaO shows a 16-fold increase in the nodules relative to its low levels in the surrounding matrix; this presumed ionic migration is sufficient to account for the carbonate content of 28% in the nodules, relative to less than 1% in the surrounding reworked tuffs. The greatly increased CO_2_ content of the nodules suggests that much of the dissolved, migrating Ca crystallised out as CaCO_3_—an interpretation that was supported by the subsequent examination of petrological thin-sections, and explains the presence in the nodules of plant fossils showing good-quality calcareous permineralisation.

However, despite the dominantly calcitic permineralisation, extensive acetate peeling of plant-containing nodules demonstrated that the anatomical detail that can be observed in acetate peels following etching in 5% HCl alone is more limited than that evident in petrological thin-sections (Bateman, 1991; Bateman, 1999). We suspect that this constraint partly reflects the comparatively low level of carbonate present in the Loch Humphrey Burn nodules (28% relative to, for example, 62% in the more readily peeled permineralised nodules from the Middle Jurassic of Skye analysed by Bateman, 1999). Also problematic is the relatively high porosity of both the matrix and the nodules at Loch Humphrey Burn, which helps to explain their unusually high values for retained water and loss on ignition (Table 2).

## Composition of the Kilpatrick Hills Macrofloras

### Glenarbuck

Glenarbuck has yielded a flora of at least nine whole-plant species that is relatively egalitarian at higher taxonomic levels: the organ-species contain the components of at least two rhizomorphic lycopsids (*Paralycopodites*, *Lepidophloios*), one sphenopsid (*Protocalamites*), three ferns (two zygopterids—*Metaclepsydropsis*, *Metadineuron*—and one coenopterid—*Botryopteris*), and two probable lyginopterid pteridosperms (*Heterangium*, *Endoxylon*), together with the much-discussed, but still taxonomically ambiguous, putative heterosporous lycopsid *Mittagia*.

Arguably the main palaeobotanical interest of the site lies in its apparently endemic species of rhizomorphic lycopsids. Comparison of the taxa documented by Smith (1962c) (incorporated in Table 1A) with the morphological phylogeny generated by Bateman, DiMichele & Willard (1992, their Fig. 3) suggests that the Glenarbuck assemblage spans the entire genus-level diversity of the clade, from the earliest-diverging characteristically Carboniferous genus *Paralycopodites* through to the most evolutionarily derived generic pairing, namely *Lepidodendron* plus the seed-plant analogue *Lepidophloios*. Although the identities of the several vegetative specimens of *Lepidophloios*, and especially of the two vegetative specimens of *‘Lepidodendron’*, are both questionable, the generic assignment of the *Lepidocarpon* specimens (the cone of the *Lepidophloios* tree) appears convincing. These records of rhizomorphic lycopsids from putatively mid-Visean Glenarbuck precede the better-established late Visean occurrences of these genera at Pettycur (Fig. 2).

### Loch Humphrey Burn: sources and ages of palaeobotanical materials

Our research has shown that previous palaeobotanists studying materials from Loch Humphrey Burn have together collected from four localities that are distributed across three different stratigraphical levels. Scott (1899, *et seq.*) studied the first-found flora, which we believe originated from the lower part of Unit 1 (the supposed late Tournaisian sequence) at Exposure A2 (Flora 1 of this study). Of the three stem-species described by DH Scott from this locality, one (*Calamopitys*) closely resembles our own collections from Flora 1. A second stem-species, *Bilignea* (Scott, 1924), was apparently attributed to this bed by Walton (1949b) and Walton (1949c), and it seems reasonable to assume that the third, *Eristophyton* (Scott, 1902), was also sourced from the same bed. However, there is no direct evidence for this sourcing; indeed, *Cleal* (in Bateman & Cleal, 1995a) tentatively suggested an origin in Unit 4. For now, the *Bilignea* and *E. fasciculare* stems must formally be regarded as unprovenanced within the Loch Humphrey Burn locality.

Scott, Galtier & Clayton (1985) collected detrital permineralisations from a nodular horizon higher in the supposedly Tournaisian succession within Exposure B (Unit 2, Flora 2). Walton and co-workers (1938, *et seq.*) obtained diverse, internationally important compressions and nodular permineralisations from the mid-Visean Unit 4 at Exposure A1 (Flora 4), and very similar material was collected from a putatively corresponding stratigraphic level at Exposure B by Scott, Galtier & Clayton (1985).

Palaeobotanically, this study has concentrated on Floras 1 and 4. Flora 1, unknown since the end of the 19th century until rediscovered by RMB and R Gray in 1985, was extensively collected. Unfortunately, all of the numerous wood specimens that were found proved to be poorly preserved. Flora 3 was first discovered and collected at Exposure B by AC Scott, and additional material of this flora was collected from the classic horizon at Exposure A1 by RMB and G Barrass. Although better preserved than Flora 1, Flora 3 is prone to fragmentation, due to the close-spaced jointing of the sediments; also, its permineralisations are both very localised and relatively inaccessible. Nonetheless, Walton’s Flora 4 remains the jewel in the crown of the remarkable fossil floras of the Kilpatrick Hills.

### Loch Humphrey Burn lower floras: Unit 1 and especially Unit 2

Irrespective of the contentious respective ages of the three beds exposed at Loch Humphrey Burn that have yielded anatomically preserved plants (especially the lower), there is no doubt that the floras are radically different in taxonomic composition, even at class level; current evidence suggests that only one whole-plant species—that most commonly represented by the stem-species *Protocalamites goeppertii*—is shared between Units 2 and 4 (Table 1).

The only plant definitely attributed to the permineralised wood assemblage of Unit 1 is the calamopitid pteridosperm stem *Calamopitys*, though the unprovenanced putative lyginopterid pterodsperm stems *Eristophyton* and *Bilignea* are also tentatively attributed to this flora.

The permineralised flora of Unit 2 contains the remains of a minimum of nine whole-plant species and most likely represents 11. Reproductive organs provide strong evidence of the presence of two species of the sphenopsid *Protocalamites*. Also found have been the lycopsid megaspore-genera Lagenicula and Setosisporites (Hemsley, Galtier & Clayton, 1994). Most diverse are the ferns *sensu lato*. Vegetative remains translate into four genera (*Cladoxylon*, *Clepsydropsis*, *Metaclepsydropsis*, *Botryopteris*) representing three orders *sensu* Stewart & Rothwell (1993), though the sporangial assemblages imply the presence of the earliest known Marattialean fern (Meyer-Berthaud & Galtier, 1986) and perhaps two additional fern species representing the Tedeleaceae and Corynepteridaceae respectively (Galtier & Scott, 1985; Scott, Galtier & Clayton, 1985; Scott & Galtier, 1996). This is a tantalising assemblage from the viewpoint of macrofossil-mediated stratigraphic dating, as it combines taxonomic groups characteristic of the late Devonian and Tournaisian (notably *Cladoxylon*) with others that are more typical of the Visean and Pennsylvanian (e.g., Marattiales), and shows especially strong genus-level similarity with the late Visean flora at Pettycur. Not surprisingly, these two floras have figured especially prominently in discussions of the early diversification of the phylogenetically heterogeneous “ferns” (e.g., Galtier & Scott, 1985).

Gymnosperm diversity is much lower than fern diversity in these Units. Lyginopterids are represented only by a single petiole that cannot be attributed with confidence to a whole-plant genus. In contrast, there are abundant remains of at least one calamopitid pteridosperm, *Calamopitys*, though they are insufficiently well preserved to provide detailed information regarding the biology of the source plant.

### Loch Humphrey Burn upper floras: Unit 3 and especially Unit 4

The upper flora from Loch Humphrey Burn contains a minimum of six (more likely seven) whole-plant species. Its composition, in terms of higher taxa, is more typical of permineralised Mississippian assemblages of the Midland Valley, being dominated—in terms of both species diversity and biomass—by pteridosperms (cf. Scott, Galtier & Clayton, 1984; Galtier & Meyer-Berthaud, 2006). It has yielded only one fern, *Etapteris*, and only one sphenopsid, *Protocalamites.* However, in combination with material from Oxroad Bay in East Lothian, the presence of both vegetative and reproductive remains of *Protocalamites* in Unit 4 has allowed much better understanding of the biology of the Archaeocalamitaceae and elucidated its place in sphenopsid phylogeny (Bateman, 1991; Rothwell & Nixon, 2006).

This flora has similarly yielded only one progymnosperm, *Protopitys*, but this species has been critical in circumscribing the order Protopityales (e.g., Beck & Wight, 1988; Decombeix, Galtier & Prestianni, 2015), due to the availability at Loch Humphrey Burn of material that reveals the external morphology, vegetative and reproductive anatomy of portions of the plant (Walton, 1957; Smith, 1962b). These data have in turn enhanced our knowledge of the phylogeny of progymnosperms (Hammond, 2004; Hilton & Bateman, 2006), while the contents of the sporangia have informed conceptual discussions of the evolution of heterospory (Bateman & DiMichele, 1994).

The most diverse and most abundant group in the flora is the pteridosperms, though there is a dearth of permineralised stem-species relative to permineralised reproductive organs and compressed foliage; this lacuna may have prompted the tentative assignment of the unprovenanced *Bilignea resinosa* and *Endoxylon fasciculare* to Unit 4 by Cleal & Thomas (1995). In addition, the evidence for the presence of a calamopitid is, as in Unit 2, confined to a petiole and microsporangia. Among the lyginopterids, the stem-species *Eristophyton waltonii* certainly forms the basis of a whole-plant taxon, but its relationship with *E. fasciculare* is unclear; they could simply constitute different ontogenetic stages of the same whole-plant species (cf. Speck & Rowe, 1994). Both species are readily distinguished from *Bilignea* (Bateman & Rothwell, 1990; Seyfullah, 2009).

Correlations among the different categories of permineralised organ are instructive, since among the putative lyginopterids there are three species of stem/branch, three species of petiole, and three species of ovulate cupule. The latter (*Calathospermum*, *Geminitheca* and, more recently, cf. *Pullaritheca*) are particularly morphologically diverse, and have strongly influenced thinking on homologies with the female organs of more derived gymnosperms, as well as prompting the concept of a strongly multi-ovulate “megacupule” (e.g., Long, 1977). This cupule assemblage has a striking parallel in the more species-rich pteridosperm assemblages from the late Tournaisian of Oxroad Bay where, for example, the co-occurrence of *Bilignea* stems with *Pullaritheca* cupules is evident (Bateman & Rothwell, 1990; Bateman & Hilton, 2009).

Comparison of the permineralisation and compression organ-species (especially the fern-like foliar organs) is also instructive when attempting to estimate the whole-plant diversity of the flora (Tables 1C and 1D). However, there is an expectation that leaf-species will on average outnumber species of reproductive organ, since the different morphologies associated with early versus late developmental stages of leaves, or so-called shade versus sun leaves, of the same whole-plant species tend to result in over-splitting when delimiting organ-species of leaves. This is especially true of compressions (note, for example, the earlier recognition at Loch Humphrey Burn of four compression species of *Rhacopteris*, two reportedly in abundance: Kidston, 1923–1925).

A close relationship between the two dimensional *Archaeocalamites* and anatomically-preserved *Protocalamites* has become widely recognised (e.g., Bateman, 1991). Cleal & Thomas (1995) tentatively assigned the foliage genus *Rhodeopteridium* to the true ferns, thereby providing a potential compressed correlate for the permineralised *Etapteris*. However, a cautionary tale is provided by the fact that Cleal & Thomas also argued that the foliar genus *Rhacopteris*, unusual in its non-forking petiole and webbed pinnae, resembles the foliage of the classic late Devonian progymnosperm *Archaeopteris* and hence may correlate with the partially permineralised *Protopitys*. This hypothesis was undermined first by the recognition that these small, distinctive fronds bear not only microsporangia (Walton, 1926) but also ovulate cupules (Vega & Archangelsky, 1996), and later by the discovery at Weaklaw, East Lothian of leaves that are partially compressed and partially permineralised, thereby revealing the petioles of both *Rhacopteris* and *Spathulopteris* to be assignable to *Lyginorachis* (Galtier, Meyer-Berthaud & Brown, 1997). This observation suggested that the plants that bore these fronds were lyginopterid pteridosperms. Noting the similarity of the Loch Humphrey Burn petioles to those found attached to *Bilignea* branches at Oxroad Bay by Bateman & Rothwell (1990), Galtier, Meyer-Berthaud & Brown (1997) tentatively suggested that *Bilignea* plants bore *Rhacopteris* leaves; these authors further suggested that these leaves were confined by developmental constraints to the apices of the subtending branches. Thus was a whole-plant concept further developed and a salutary warning simultaneously given regarding the dangers inherent when invoking overly speculative correlations of organ-species (cf. Bateman & Hilton, 2009). We therefore view as tentative our suggested taxonomic affinities of the compression leaf-species of *Spathulopteris*, *Sphenopteridium* and *Sphenopteris* recorded from Loch Humphrey Burn (Table 1D).

Nonetheless, Loch Humphrey Burn is one of the few Mississippian localities to yield well-preserved plant fossils that combine the preservation states of permineralisation and compression, thereby offering the potential for correlating features of anatomy and gross morphology, together with information on any microspores or megaspores present. Such preservation considerably enhanced the value of specimens in permineralised nodules extracted from Unit 4, such as *Protopitys*, *Alcicornopteris*, *Staphylotheca*, *Calathospermum* and, more recently, cf. *Pullaritheca* (Fig. 8, Table 1).

To summarise the contents of the less thoroughly investigated compression floras, Unit 3 appears similar in species composition to the better researched Unit 4, being dominated by presumed pteridosperm (and possibly progymnosperm) foliage. In contrast, the limited palaeobotanical material extracted from Unit 5 indicates domination by arborescent lycopsids, and thus more closely resembles the Glenarbuck flora (see ‘Stratigraphy and palaeoecology of the Kilpatrick Hills floras’ and ‘Floristic comparison of Mississippian lagerstätten from the Scottish Midland Valley and Tweed Sub-basin’).

### Summary of macrofossil diversity through the Loch Humphrey Burn sequence

Reviewing the macrofossil data previously summarised by Bateman (1986, unpublished data), Stevens (2009) estimated the number of whole-plant species of each taxonomic class (*sensu* Stewart & Rothwell, 1993) present in each of five beds within the Loch Humphrey Burn and Glenarbuck successions, including the famous Walton Bed within Unit 4 (Fig. 9). Viewed at this coarse taxonomic resolution, Unit 1 is dominated by pteridosperms, whereas Unit 2 is rich in filicopsids. The more thoroughly sampled Unit 4 is also dominated by pteridosperms but contains some filicopsids and sphenopsids, and shows the earliest macrofossil evidence of lycopsids at the site. Interestingly, the permineralisation-bearing Walton Bed appears to be more taxonomically egalitarian than do the surrounding compression-bearing sediments; it includes more filicopsids and presents good evidence of progymnosperms, but apparently lacks lycopsids. Lastly, the Glenarbuck sequence—here controversially attributed to the central portion of Unit 5—contrasts strongly with Units 1–4, being dominated by lycopsids and filicopsids (Fig. 9).

Our next task was to determine whether the microfloras of the Kilpatrick Hills yielded floristic data congruent with those already obtained from the macrofloras.

## Composition of the Kilpatrick Hills Microfloras

### Background

The palynology of fossil plant-bearing localities of the Kilpatrick Hills was first investigated by G Clayton (in Scott, Galtier & Clayton, 1984), who collected 22 samples from Glenarbuck but only seven from Loch Humphrey Burn, none of which was located in our Unit 1 and only one in our Unit 2. In 1987, SA Brindley prepared and compared single palynological samples from the nodule-bearing horizon at Lang Craigs and the nodule-bearing horizon in Unit 4 of Loch Humphrey Burn.

However, the next serious attempt to develop through palynology a better understanding of vegetational composition at Loch Humphrey Burn was made by Stevens (2009). She conducted a palynological analysis of miospores with known or suspected affinities to potential source whole-plants. This technique was applied successfully to slightly younger Pennsylvanian microfloras by Dimitrova, Cleal & Thomas (2005), Dimitrova & Cleal (2007) and Willard et al. (2007), who used palynological data to offset the taphonomic and palaeoenvironmental biases inherent in Pennsylvanian peat-forming mire macrofloras. However, previous studies of Mississippian palynology have emphasised either palynochronology or palaeoclimates rather than palaeoecology *per se* (cf. Van der Zwan, 1981; Van der Zwan, Boulter & Hubbard, 1985; Stephenson et al., 2002; Stephenson et al., 2008). Fortunately, gradually increasing knowledge of *in situ* spores (e.g., Balme, 1995) is rendering more feasible palaeoecological interpretation of Mississippian microfloras.

Compared with plant macrofossils, palynomorphs are on average produced in larger numbers, are more readily preserved, and sample a broader hinterland, irrespective of geological age (cf. Farley, 1990; Mander, Kürschner & McElwain, 2010; Looy et al., 2014). They are also generally more prone to reworking from pre-existing sediments. Both microfloral and macrofloral data are subject to biases in diversity and abundance, but when used in conjunction, they are likely to provide a more reliable picture of the patterns of change in floral composition through time (e.g., DiMichele & Hook, 1992). Similar studies conducted on younger floras (Lidgard & Crane, 1990; Lupia, Lidgard & Crane, 1999; Liang et al., 2003) found broad correlations between macrofloral and palynofloral diversity patterns, and demonstrated that the approach is most effective when exploring floristic trends at a single locality. These conclusions encouraged us to pursue such a study at Loch Humphrey Burn.

### Materials and methods

Palynological samples were collected from newly dug trenches that followed four transects previously logged by RMB (Figs. 5 and 6). About 0.5 kg of fresh sediment was taken from every horizon present in the transects, totalling 68 samples from a 30 m composite section. Processing limitations meant that only 30 finer-grained samples judged most likely to contain well-preserved spores—distributed fairly evenly across the transects—were chosen for maceration.

Palynological processing (cf. Batten, 1999) was conducted at Trinity College, Dublin, under the supervision of G Clayton. Rock samples were crushed into pea-sized chips, placed in 0.5 litre plastic containers and washed with water to remove particles finer than 20 µm. Two drops of 10% HCl were added to test for carbonate, and this treatment was repeated until effervescence ceased. Approximately 20–40 ml of 40% HF was then added, monitored initially for overheating, and then left until all siliciclastic material had dissolved. The remaining HF was decanted over CaCO_3_ powder and the samples washed with water five or six times until neutralised. The organic residue was sieved through 15 µm mesh and transferred to 10 ml glass vials. Each sample was checked for the presence of miospores; if necessary, they were lightened in colour using HNO_2_ and/or further dissolved in HCl. One drop of HCl was added to the samples to prevent algal growth during storage.

Each of the 11 samples found to contain good-quality miospore assemblages was slide-mounted in Eukitt. Identifications were made under a Zeiss Axioskop 40 microscope, using a mounted Canon EOS digital SLR camera to photograph the first 250 spores in each sample that were judged to be intact (i.e., outline entire, ornamentation clearly visible, orientation random: cf. Brindley & Spinner, 1989). If many more than 250 miospores were found in a sample, the rest of the slide was scanned to ensure that the first 250 miospores were representative of the entire assemblage.

### Diversity and local stratigraphic trends in miospore genera

The micrographic images were classified only to organ-genus level due to time constraints. Figure 10 illustrates eight miospore genera commonly found within the Loch Humphrey Burn sequence. Identifications of spore genera relied upon several monographs of Carboniferous spore assemblages (Playford, 1962; Smith & Butterworth, 1967; Kosanke, 1969; Traverse, 1988; Stephenson & Owens, 2006). The resulting data were arranged as an abundance/diversity matrix in Microsoft Excel.

The occurrence and stratigraphic distribution of spore genera are given in Table 3. In this study, relative diversity (as opposed to absolute diversity) of spores and macrofossils was calculated as a percentage of taxa compared with all the other organ-species found in that sample, in order to render the samples free from counting biases and to limit sedimentary biases (Nagalingum et al., 2002). Exploratory data analyses were performed using PAST (Hammer, Harper & Ryan, 2001), a statistical package designed explicitly for palaeontological data analysis.

**Table 3:** Distribution of 53 miospore organ-genera through 11 samples in the composite sequence at Loch Humphrey Burn. Datasets are based on counts of 250, except sample 10 which yielded only 43 identifiable miospores. The ‘Other’ category consists of miospore genera that either have been attributed to more than one taxonomic class or have not yet been credibly attributed to any class: *Anaplanisporites*, *Bacculatisporites*, *Cheilidonites*, *Cingulizonates*, *Colatisporites*, *Convolutispora*, *Discernisporites*, *Kraeuselisporites*, *Lophozonotriletes*, *Monilospora*, *Murospora*, *Polygnathus*, *Punctatisporites*, *Pustulatisporites*, *Retusotriletes*, *Rugospora*, *Schopfites*, *Spelaeotriletes*, *Spinozonotriletes*, *Stenozonotriletes*, *Tricidarisporites*, *Umbonatisporites*.

Taxonomic class	Miospore genus											
		1	2	3	4	5	6	7	8	9	10	11
Lycopsida	*Anapiculatisporites*				0.40			1.61				
Lycopsida	*Cadiospora*			0.81								
Lycopsida	*Cirratriradites*			0.40								
Lycopsida	*Crassispora*	5.22	0.42	0.81	7.60							0.41
Lycopsida	*Densosporites*		1.26	1.61		0.40	0.40	1.61				1.23
Lycopsida	*Endosporites*	0.80	5.46	3.63		0.80	0.40	2.01	2.80	0.41		0.82
Lycopsida	*Foveosporites*		0.84	0.40				0.80	0.40	1.23	4.65	1.65
Lycopsida	*Lycospora*	4.42	2.52	4.03	21.60	4.42	0.80	5.22	4.80	30.86		0.82
Lycopsida	*Planisporites*			0.40	0.40			0.80				
Lycopsida	*Radiizonates*		0.42						0.40			
Sphenopsida	*Calamospora*	20.88	7.14	16.53	38.80	13.65	18.07	13.25	13.20	18.52		20.99
Sphenopsida	*Vetispora*	1.20	0.84	0.40		1.61		1.61	4.80			1.65
Filicopsida	*Acanthotriletes*		1.26				0.40	0.40	1.20			6.17
Filicopsida	*Apiculatasporites*			0.81			4.42	4.42				
Filicopsida	*Apiculatisporis*	2.41		0.81	0.80		1.20	3.61	0.80	0.41		1.23
Filicopsida	*Camptotriletes*							0.40				1.23
Filicopsida	*Cyclogranisporites*	0.40	2.52	2.82	3.20	5.62		1.20	4.00	1.23		4.12
Filicopsida	*Dictyotriletes*		6.72	5.24		3.61	8.03	3.61	2.40	4.53		0.82
Filicopsida	*Granulatisporites*	2.01	0.84	0.81				0.40		0.41	2.33	0.41
Filicopsida	*Grumosisporites*						0.40		0.40		6.98	0.82
Filicopsida	*Knoxisporites*	3.21	9.66	9.27	0.80	6.43	5.22	4.42	6.80	4.94		6.17
Filicopsida	*Leiotriletes*	2.01	2.10	2.02	0.80	2.01	3.21	6.43		1.23		
Filicopsida	*Lophotriletes*			0.40		0.40	8.03	1.20		1.23		0.41
Filicopsida	*Microreticulatisporites*	2.81	6.30	0.81	11.20	0.80	5.22	2.81	2.80	5.35		2.88
Filicopsida	*Raistrickia*	0.40	2.10		0.40							0.41
Filicopsida	*Reticulatisporites*	3.61	0.84			2.41	7.63	3.21	3.60	1.65	13.95	0.41
Filicopsida	*Retispora*											0.41
Filicopsida	*Triquitrites*			0.40								
Filicopsida	*Verrucosisporites*	0.40	17.23	0.40	4.40			9.24	4.40	2.47	13.95	4.94
Pteridosperm	*Alatisporites*						0.40	0.40				
Pteridosperm	*Rotaspora*					0.40	0.40			0.82	2.33	1.23
NA	Other	50.20	31.51	47.18	9.60	57.43	35.74	31.33	47.20	24.69	55.81	40.74

In total, the 11 palynological samples quantitatively analysed yielded a remarkable 53 genera of miospores (Table 3): of these, 10 were tentatively assigned to Lycopsida, two to Sphenopsida, 17 to Filicopsida and, surprisingly, just two to Gymnospermopsida (i.e., pteridosperms). A further 22 miospore genera were treated as *incertae sedis*; comparison with the macrofloras suggests that the bulk of these unclassified miospore-genera probably represent pteridosperms and/or progymnosperms. Regrettably, three of these *incertae sedis* spore genera occurred at high frequencies in at least a majority of the samples analysed: *Convolutispora* (≤44%), *Punctatisporites* (≤21%) and *Stenozonotriletes* (≤6%). The first two of these three miospore-genera were tentatively attributed to pteridosperm pollen-organs described from Oxroad Bay by Bateman (1988), but *Convolutispora* species have also been found in filicopsids, and *Punctatisporites* in members of several major taxonomic groups. The sample from the Walton Bed in the centre of Unit 4 yielded the largest number of miospore genera (30), whereas the lower of the two samples analysed from within Unit 5 yielded the smallest number (10), albeit handicapped by the small number of miospores recovered.

These palynological data are viewed at the crude level of taxonomic Classes (*sensu* Stewart & Rothwell, 1993) in the left-hand of the two columns presented in Fig. 9. Lycopsid microspores are relatively frequent in the comparatively fine sediments that occur close to the bases of Unit 4 and especially Unit 5; in the former they are accompanied by a peak in sphenopsid microspores, and together these pteridophytes greatly reduce the relative amounts of pteridosperm prepollen. Filicopsid spores tend to be more frequent in the coarser sediments, but surprisingly, they also dominate the uppermost palynological sample in Unit 5, located only *ca* 3 m above palaeosols demarcated by lycopsid rootlets (Fig. 9). Admittedly, comparatively few miospores were found in this sample, which is therefore subject to greater sampling error. In terms of palynology, the Walton Bed does not differ markedly from those adjacent to it in the succession, somewhat in contrast with the patterns suggested by the macrofossil data.

Figure 11 presents a Detrended Correspondence Analysis (DCA) that simultaneously ordinated the spore genera and sample localities. The evident clustering of assemblages corresponds well with the palynoflora histogram shown in Fig. 9. Axis 1 separates the most lycopsid-rich assemblages (samples 1, 3–5, 10) from the most sphenopsid-poor assemblages (samples 2, 11). The much weaker Axis 2 is dictated primarily by contrasting suites of subordinate (in most cases less than 1%) spore-genera, mostly filicopsids. The three samples scoring lowest on Axis 2 contain traces of *Acanthotriletes* (samples 2, 8 and 9), *Radiizonates* (samples 2, 9), *Raistrickia* (sample 2), *Camptotriletes* and *Retispora* (both sample 8). In contrast, the three samples scoring highest on Axis 2 contain significant proportions (>5%) of otherwise rare filicopsid spore-genera, either *Apiculatasporites* (samples 6 and 7) or *Grumosisporites* (sample 11). Axis 3 (not shown) merely separates sample 7 from the remainder, largely on the basis of the presence of small quantities (*ca* 2%) of the lycopsid spore-genus *Anapiculatisporites*. Only occasionally do stratigraphically adjacent samples plot close to each other in Fig. 11 (notably 3 plus 4 and 8 plus 9), indicating frequent fluctuations in relative proportions of spore-genera.

### Comparison of the microfloral and macrofloral trends

The microfloral results show considerably less variation in genus-level composition across the sequence than do the macrofossils (Fig. 9). This observation is likely to partly reflect reduced sampling error permitted by larger sample sizes inherent in palynological studies, but is probably largely explained by the fact that (unsurprisingly, given their explicit dispersal function) miospores are comparatively easily transported by wind as well as water, and so generally sample a wider catchment. As a sweeping generalisation, we suspect that in the Kilpatrick Hills, the microfossils sampled the regional flora and the macrofossils sampled the local flora.

The relative consistency of the miospore data suggests that the gymnosperm-only macroflora of Unit 1 (exclusively woody stems and branches) is highly unrepresentative of the regional vegetation, and is almost certainly a taphonomic ‘artefact’ of sorting in these coarse, high-energy sediments. Foliar and reproductive organs of pteridosperms are the most frequent macrofossils in the other high-energy, volcaniclastic-rich portion of the succession, Unit 4, but here again, this trend is not reflected in the microfossil assemblages. Also, sufficient contrast is evident between the macrofossil dataset gathered from the Walton Bed in the middle of Unit 4 relative to the dataset generalised across the whole of Unit 4 to suggest that finer-scale sampling of the macrofloras would have been advantageous. Both microfossil and macrofossil datasets agree that the upper part of Unit 5 is comparatively rich in ferns, but the increase in lycopsids suggested by the macrofossil data is not mirrored in the microfossils (admittedly, the relevant samples were separated by a substantial stratigraphic distance of *ca* 3 m).

However, the most striking incongruity that runs through the entire sequence is the lower frequency of pteridosperms in the microfloras compared with the macrofloras (Fig. 9). Similar observations have been made in previous studies of slightly younger, Pennsylvanian sequences (e.g., DiMichele, Phillips & Pfefferkorn, 2006; Bashforth, Falcon-Lang & Gibling, 2010), tentatively suggesting that pteridosperms may have reproduced less frequently and/or generated smaller numbers of miospores per plant than other coeval plant groups. Alternatively, pteridosperms may have eschewed unstable habitats such as those prevailing at Loch Humphrey Burn. Admittedly, these conclusions were drawn by previous authors primarily from studies of Pennsylvanian medullosan pteridosperms, which were highly apomorphic in reproductive characteristics and lack obvious comparators in the earlier Mississippian floras of Scotland. Moreover, habitat disturbance did not prevent pteridosperms from dominating Scottish Mississippian plant communities such as those described in detail from the Upper Tournaisian of Oxroad Bay (Bateman & Rothwell, 1990; Bateman & Scott, 1990).

## Stratigraphy and Palaeoecology of the Kilpatrick Hills Floras

### Stratigraphic summary

The 30 m sequence recorded at Loch Humphrey Burn that is described in detail on pages 21–26 and summarised in Fig. 7 is composed almost entirely of fluvial sediments; coarse channel deposits (Units 1 and 4) alternate with finer flood-plain sediments (Units 2, 3 and 5). Unit 2 also contains thin lacustrine deposits. Much of the sediment was derived from the surrounding volcanigenic terrain. Organic input varied in quantity, quality and botanical affinities. In terms of local biomass estimated from the macrofloras, Units 1–4 mainly yield allochthonous pteridosperms, but Unit 5 largely contains autochthonous or hypoautochthonous lycopsids. Syndepositional cementation played a key role in preserving the plant materials, some of which are of exceptional quality.

### Palynochronology

Relative dates derived from miospore assemblages have been interpreted as indicating that the Loch Humphrey Burn sequence is very condensed, and presumably includes at least one substantial gap. Most notably, Pu palynozone assemblages, which characterise up to 90 m of sediment further east (Neves et al., 1973), have at Loch Humphrey Burn only been reported in the 20 cm-thick ‘Fish Bed’ (located within our Unit 2). This startling observation prompted Scott, Galtier & Clayton (1984) and Scott, Galtier & Clayton (1985) to suggest that the ‘Fish Bed’ marks a considerable break in sedimentation, even though there is no obvious erosion surface, either below or above this distinctive bed. However, miospores have been examined from only one other bed in Units 1–3; specifically, the permineralisation-bearing ‘Bed 6’ of Scott, Galtier & Clayton (1984), located at the base of our Unit 2, which was controversially dated as CM palynozone (late Tournaisian). Miospore assemblages spanning the plant-rich Walton Bed (our Unit 4) were initially described as “indicating a Pu Zone age with some elements of the TC Zone” (Clayton et al., 1978; Scott, Galtier & Clayton, 1984), though this assemblage was later used as the basis to establish the intervening TS Zone (Clayton, 1985) (Fig. 2). Therefore, on palynological evidence:

(1) Unit 1, which contains an important flora (see ‘Unit 1: Green-grey ashy conglomerates’), must be CM palynozone *or earlier* (i.e., Hastarian–Ivorian: late Tournaisian);
(2) The 1.5 m of sediment between the top of ‘Bed 6’ (which contains another important flora) and the base of ‘Bed 11’ (the ‘Fish Bed’) of *Scott, Galtier & Clayton* (i.e., the bulk of our Unit 2) could be CM palynozone (Ivorian: late Tournaisian) or Pu palynozone (Chadian–Arundian: early Visean);
(3) The *ca* 3.65 m of dark grey shales that comprise Unit 3 could be Pu palynozone (Chadian–Arundian: early Visean) or TS palynozone (Holkerian: mid-Visean);
(4) The plant-rich Unit 4 is confidently attributed to the TS palynozone (Holkerian: mid-Visean), presumably along with the lycopsid-dominated palaeosols of Unit 5. The Kilpatrick Hills are *de facto* the *locus classicus* for this palynozone.

AC Scott and colleagues later expressed considerable confidence than their earlier and more tentative conclusions: “This new research, which included more precise dating of the differing plant bearing levels, indicated that there were at least two floras of different ages (a new[ly found] late Tournaisian flora and a mid-Viséan flora). Subsequent palynological work indicated that the age of the Upper flora could be revised yet further to an early Upper Viséan age (Scott, 1990). The history of this flora indicates a major potential source of error in the age assignment of (fossil) floras which have not been personally studied by the palaeobiogeographer” (Scott & Galtier, 1996, p. 150).

However, Bateman (1986, unpublished data) noted that “if Pu miospore assemblages do prove to be confined to the ‘Fish Bed’, this interval is inferred to represent *ca* 8 my of deposition between the late Tournaisian sediments below and the mid-Visean sediments above. Although this lacustrine bed presumably represents a more quiescent period of deposition compared with the rapidly fluctuating fluvial sedimentation above and below, it seems extremely unlikely that either (a) it would take *ca* 8 my for 20 cm of sediment to accumulate, or (b) such a long period of non-deposition could occur in a tectonically active terrestrial environment without detectable erosion taking place. It is more probable that the palynozones, most of which were originally established for Mississippian sediments in eastern Scotland, require revision and refining when they are applied to sediments located further west.” Indeed, based on these miospore dates, the entire section at Loch Humphrey Burn is inferred to represent *ca* 14 Myr (Fig. 2).

Unsurprisingly, the miospore dates first published by Scott, Galtier & Clayton (1984) (especially the CM Zone age attributed to Unit 1) have caused consternation among both stratigraphers and evolutionary palaeobotanists ever since (reviewed by Monaghan & Browne, 2010). As noted by Monaghan & Parrish (2006, p. 22), “the Loch Humphrey Burn CM zonation [reported] from well-preserved palynomorphs (Scott, Galtier & Clayton, 1984) in the Greenside Volcaniclastic Member is difficult to explain when compared with the younger Pu zonation of the underlying Clyde Sandstone Formation in the nearby borehole”, particularly as “the Kirkwood and Lawmuir formations (Strathclyde Group) and Lower Limestone Formation (Clackmannan Group) diachronously overlie the Clyde Plateau Volcanic Formation and have yielded NM–VF miospore zone determinations (top Asbian–Brigantian; BGS Kirkwood and Lawmuir boreholes: Owens, 1980b)” (compare with the present Fig. 2). Monaghan & Parrish (2006) argued that the Greenside Volcaniclastic Member should be “taken as Pu–TS miospore zonation, and that “the CM zonation (Scott, Galtier & Clayton, 1984; Scott, 1990) [should be] ignored”. Understandably, they concluded that “the difficulties with palynological zonations within and below the [Clyde Plateau Volcanic] Formation mean that further dating work in the northern [= Kilpatrick and Campsie] fault blocks would be beneficial”, and that “further work on palynomorph determinations and isotopic dates from key sites such as the Greenside Volcaniclastic Member at Loch Humphrey would be useful, so that the Midland Valley of Scotland Visean numerical (radiometric) dates can be more confidently assigned to stage level” (Monaghan & Parrish, 2006, pp. 23, 26). We agree.

Examining this conundrum in greater detail demonstrates that several unfortunate factors have conspired to make dating plant-bearing sequences in the Kilpatrick Hills especially problematic. Firstly, when correlated with the absolute time-scale of Menning et al. (2000), the crucial Pu Zone is perceived as being by far the longest of the Mississippian miospore zones (Fig. 2). Similarly, the corresponding (but less resolved) plant macrofossil zone (Mp2 *sensu* Wagner, 1984) is also unusually long, spanning the *ca* 20 Myr period that happens to coincide with the entire spectrum of potential pollen dates attributed to the combined Loch Humphrey Burn and Glenarbuck sequence (Fig. 2). Lastly, the miospore age estimates for Glenarbuck and Long Humphrey Burn are both extraordinarily long, the former because the analysed assemblages were poorly preserved and hence difficult to identify to organ-species level, and the latter because they were strongly internally contradictory.

We will return to this important topic in ‘Palaeoecology’, after briefly reconsidering aspects of the available lithostratigraphic evidence.

### Lithostratigraphic correlation

In addition to Glenarbuck and Loch Humphrey Burn, several other exposures of sub-laval “tuffs and agglomerates” in the Kilpatrick Hills examined during the present study revealed laminated greenish-brown siltstones and fine sands exhibiting several thin impersistent black organic shales and poorly developed palaeosols delimited by *in situ* stigmarian rootlets of arborescent lycopsids (introduced in ‘History of palaeobotanical exploration in the Kilpatrick Hills’). Bateman (1986, unpublished manuscript) concluded that “these exposures are therefore identical with, and probably correlate with, the lycopsid-dominated uppermost beds (Unit 5) at Loch Humphrey Burn. This suggests that either (a) Units 1–4 are very localised, or (b) they are widespread but rarely exposed.”

Bateman concluded that “geomorphological evidence supports the latter hypothesis.” Specifically, he noted that at most other palaeobotanical sites in the heavily glacially eroded Kilpatrick Hills, including Loch Humphrey Burn and Glenarbuck (Fig. 3), plant-bearing rocks crop out immediately below lava scarps and are probably exposed through erosion caused by run-off from the relatively impermeable lavas. Lower parts of the sub-laval sediments are routinely obscured by glacial till and/or post-glacial talus. Most such exposures have developed along faults that approximately coincide with the main lava scarp, including Glenarbuck (Fig. 12). The Loch Humphrey Burn exposures also occur along a fault, but this does not coincide precisely with the lava scarp (Fig. 13); moreover, the lava scarp is subdued and set back from the main topographic scarp, which is formed of sub-laval sediments. A stream has cut back into this scarp as far as the lavas, forming a miniature ‘hanging valley’ that is bounded at either end by waterfalls (Figs. 4A and 4C) and exposes a greater thickness of sediment than can readily be examined elsewhere in the Kilpatrick Hills. It therefore appeared possible that the lycopsid-dominated Unit 5 was underlain by the pteridosperm-dominated Unit 4 over much of the Kilpatrick Hills.

Although studies of stratigraphy in the Hills based on borehole data and lava lithology permits the correlation suggested by Bateman (1986, unpublished manuscript) between Unit 5 at Loch Humphrey Burn and the entire Glenarbuck sequence, the sediments enclosing both sequences appear to be intercalated within the series of basaltic lavas that cap the Hills. In contrast, the majority of the other exposures, such as Lang Craigs (Fig. 3), appear to immediately underlie the lowermost of the lavas, and thus are presumed to be older than the similar strata observed at Loch Humphrey Burn and Glenarbuck (this interpretation assumes that the lowermost lava extends east–west across a distance of at least 2 km). This conclusion opens the way for the following, ecologically based, re-interpretation of the stratigraphic and dating challenges still posed by these floras.

### Palaeoecology

The most parsimonious synthesis of the disparate information assembled here is as follows. The TS palynozone attributed to Units 4 and 5 at Loch Humphrey Burn is correct. The Glenarbuck sequence is here equated with Unit 5 at Loch Humphrey Burn, a correlation permitted by the broad date of “Pu palynozone *or younger*” (Scott, Galtier & Clayton, 1984), since the TS zone immediately succeeds the Pu zone. Correlation of the TS zone with the Holkerian substage (mid-Visean) is consistent with the high-precision radiometric dates obtained from the Clyde Plateau lavas (summarised by Monaghan & Parrish, 2006).

The only remaining incongruent elements are the single CM zone miospore assemblage from the base of Unit 2 and the Pu zone miospore assemblage from the base of Unit 3 reported for Exposure B at Loch Humphrey Burn by Scott, Galtier & Clayton (1984). These discordant dates could conceivably be explained by reworking of miospores from older sediments, but this explanation seems especially unlikely if the Glenarbuck and Loch Humphrey Burn sequences are indeed sandwiched within extensive lava flows. A more credible explanation invokes local palaeoecology, revisiting the floristic hypotheses of Smith (1960; see also Smith, 1964) and Scott, Galtier & Clayton (1984) in the light of more recent stratigraphic information.

Specifically, our impression gained from fieldwork across the region is that the typical climax vegetation, both immediately before and during the eruption of the lavas, was clastic swamps that occasionally allowed modest accumulation of sediment richer in plant debris (i.e., ‘peat’ *sensu latissimo*). These communities were dominated by lycopsid trees with an interstitial flora that included horsetails, plus a few specialist ferns and pteridosperms. In contrast, Units 1–4 at Loch Humphrey Burn record a rapidly changing series of communities dominated in terms of biomass by pteridosperms and progymnosperms, but with at least sporadic understorey vegetation that contained seemingly rotating suites of several fern species but retained the persistent and ecologically adaptable horsetails throughout. Occasional wildfires are evident, especially in Units 1 and 2. These fires could have benefited from the gradual elevation of atmospheric oxygen levels during the late Palaeozoic, though current estimates suggest that oxygen levels in the mid-Visean were only slightly higher than those of today, rising to a peak of *ca* 32 ± 2% only in the Permian (cf. Karhu & Holland, 1996; Berner, 1997; Scott & Glasspool, 2006; Glasspool et al., 2015). It has become conventional wisdom that such disturbed communities characterise volcanigenic landscapes throughout the Mississippian (Bateman & Scott, 1990; Scott, 1990).

**Table 4:** Comparison of Mississippian macrofloras. Number of organ-species of each major taxonomic group of vascular land-plants occurring in 15 anatomically preserved floras in the Mississippian of southern Scotland, northern England and the French Massif Central. Localities listed from northwest to southeast.

Locality	Age	Sphenopsids	Filicopsids	Progymnosperms	Gymnosperms	Lycopsids	TOTAL
Kilpatrick (Unit 2)	??L. Tournaisian	5	12	0	6	0	23
Kilpatrick (Unit 4)	M. Visean	6	4	1	27	2	40
Kilpatrick (Unit 5)	?E.-L. Visean	1	3	0	5	4	13
East Kirkton	L. Visean	3	1	1	14	6	25
Pettycur	L. Visean	4	4	0	3	6	16
Castleton Bay	L. Tournaisian	0	2	1	2	4	9
Oxroad Bay	L. Tournaisian	2	2	0	28	3	35
Cove	L. Tournaisian	0	1	0	10	1	12
Langton	L. Tournaisian	0	3	0	15	1	19
Edrom	L. Tournaisian	0	4	0	21	2	27
Foulden	L. Tournaisian	0	0	0	3	0	3
Burnmouth	L. Tournaisian	0	1	0	3	1	5
Esnost	L. Visean	2	5	0	0	3	10
Roannais	L. Visean	0	3	0	0	1	4
Montagne Noire	M.-L. Tournaisian	0	4	0	8	2	14

The key question in the context of the present paper is whether these rapid shifts in communities could account for the anomalously early palynozone identifications attributed to Units 2 and 3 by Scott, Galtier & Clayton (1984). The idea at least merits further exploration, along with consideration of the possible influences on the regional vegetation of contemporaneous climate change. Unfortunately, active volcanicity can have a profound impact on perceptions of local climate, acting both directly through processes such as ash-induced increases in cloud cover and/or rainfall and indirectly through unusual depositional process such as mass-flow (e.g., Bateman & Scott, 1990). WA DiMichele (pers. comm., 2015) tentatively interpreted the palaeoenvironments described here as reflecting a fairly arid (or at least strongly seasonal) climate. In this scenario, occasional torrential rain and flash floods occurred within a basin of limited areal extent and were interspersed with dry seasons that allowed pedogenic formation of the calcium carbonate concretions (see also Boucot, Chen & Scotese, 2013; Cecil & Brezinski, 2013; Cecil, 2015).

## Floristic Comparison of Mississippian Lagerstätten from the Scottish Midland Valley and Tweed Sub-basin

In a further attempt to gather circumstantial evidence toward dating the Kilpatrick Hills lagerstätte, Stevens (2009) compiled a data-matrix (Table 4) summarising the macrofossil compositions of organ-species recorded from Units 2, 4 and 5 of the Kilpatrick Hills with those of nine permineralised macrofloras from the eastern part of the Scottish Midland Valley and the Tweed Sub-basin (Fig. 1) plus three localities in south-central France. Organ-species compositions for most of these localities were abstracted from Scott, Galtier & Clayton (1984), supplemented with additional data for Oxroad Bay (Bateman & Rothwell, 1990; Bateman & Scott, 1990) and with the results of more recent studies conducted on new localities in Mid-Lothian (Brown, Scott & Jones, 1994; Scott & Galtier, 1994; Scott et al., 1994) and East Lothian (Galtier & Scott, 1990). Relationships among these permineralised floras were calculated in Genstat v14 (Payne et al., 2011) using the Bray–Curtis dissimilarity measure, which is influenced by both taxonomic composition and overall taxonomic diversity. The resulting dendrogram (Fig. 14) should be interpreted with caution, most notably because a single conceptual whole-plant species is often represented by two or more Linnean binomials representing organ-species derived from different parts of the same source plant (Bateman & Hilton, 2009).

Both overall organ-species diversity and class-level taxonomic composition have clearly influenced the tree topology. Three of the four most divergent floras (Roannais, Foulden, Burnmouth) are those containing the smallest recorded numbers of organ-species (Table 4), and are therefore most vulnerable to sampling error. Also, Bray–Curtis similarities are almost equal in the central portion of the tree, approximating 73–78%. Crucially, the three Kilpatrick Hills floras are distributed across the entire tree. The middle unit (4) is most similar to the late Tournaisian flora from Oxroad Bay, the two being drawn together by their exceptional numbers of pteridosperm-derived organ-species. In contrast, the lowest Kilpatrick Hills Unit (2) is the most divergent of all the floras analysed. It is placed in close proximilty to the Roannais flora from the late Visean of France, the two localities containing the largest number of organ-species derived from ferns s.l. The uppermost Kilpatrick Hills Unit (5) shows an intermediate degree of divergence, occurring among the central cluster of broadly similar floras. It is placed closest to other Scottish Visean localities at Pettycur and East Kirkton (the two localities containing the largest number of clubmoss organ-species). There is also limited evidence of geographic clustering, though two of the pairings (Edrom plus Langton, Foulden plus Burnmouth) are nearest neighbours (Fig. 1). But again importantly, the distribution of Visean versus Tournaisian floras across the dendrogram does not deviate from random.

With regard to dating the three Kilpatrick Units, it could be argued that the two Units widely regarded as being Visean in age are more similar to each other than either is to the lowermost, supposedly late Tournaisian, Unit 2. However, it is the central Unit 4 that occurs within a cluster of five indisputably late Tournaisian floras, even though stratigraphically it occurs between Units 2 and 5, which are closely associated with both Tournaisian and Visean floras (Fig. 14). We suggest that, when analysed at this relatively crude level of taxonomic resolution, the placements of fossil macrofloras in Fig. 14 are best interpreted in terms of both (a) contrasting plant communities reflecting changing local environments, and (b) contrasting subsequent taphonomic histories of each assemblage. They are not competent to confidently determine precise geological age.

## Further Palaeobotanical Research in the Kilpatrick Hills

This interdisciplinary study has addressed a wide range of scientific questions, generating strong answers to some and tentative answers to others, but also raising yet more questions. Areas demanding further research are thus both numerous and diverse:

(1) Thorough study of the large volumes of compression fossils extracted from the Loch Humphrey Burn locality could not only clarify the circumscription and taxonomic affinities of the organ-species present but also lead to a better understanding of the palaeoecological transitions that are undoubtedly captured in this sequence.
(2) Although the permineralised floras from Loch Humphrey Burn have been more thoroughly studied than the co-occurring compressions, there remains considerable potential for strengthening the existing tentative correlations of key organ-species across preservational states (cf. Galtier, 1986; Bateman & Rothwell, 1990; Bateman & Hilton, 2009).
(3) Palaeobotanical data from the Kilpatrick Hills could be more effectively integrated with information from other Mississippian localities in the Scottish Midland Valley. For example, the best opportunity to fully reconstruct the *Bilignea* plant—clearly an ecological dominant of the period—lies in combining data from Loch Humphrey Burn (present study), East Kirkton (Galtier & Scott, 1994), Weaklaw (Galtier, Meyer-Berthaud & Brown, 1997), Kingswood (Meyer-Berthaud, 1990) and Oxroad Bay (Bateman & Rothwell, 1990; Seyfullah, 2009).
(4) The continuing uncertainty regarding the stratigraphic origin of two of the organ-species of pteridosperm stems studied by Scott (1902) and Scott (1924) could be addressed by comparing their geochemistry with that of each possible source horizon—an approach previously employed successfully at Oxroad Bay (in our opinion, geochemical techniques remain under-utilised in palaeobotanical investigations: cf. Bateman, 1988; Bateman & Scott, 1990; Bateman, 1999).
(5) Sampling of miospore assemblages from various lycopsid-dominated outcrops immediately below the lowermost Clyde Plateau lavas would be useful, to challenge the supposed CM date of the overlying volcanigenic sediments, and particularly to test our prediction that the subvolcanic sediments should straddle the Pu–TS palynozone boundary (Fig. 2).
(6) Our interpretation of the complex stratigraphy of the Kilpatrick Hills has depended on radiometric dates from structural blocks to the southwest; it is highly desirable that such dates are also obtained in the Kilpatrick Hills Block—specifically, from lavas both below and above the Loch Humphrey Burn sequence. The volcanic stratigraphy of the area might also be further refined through comparison of the detailed geochemistry of successive lava flows.
(7) Given the substantial number of palaeobotanical localities already identified in the Kilpatrick Hills, we suspect that a more intensive field survey of the region would reveal additional, potentially valuable, plant-bearing outcrops.

In conclusion, further work toward reconstructing conceptual whole-plants would allow the Loch Humphrey Burn pteridosperms in particular to be explored in the wider context of broad-brush morphological phylogenetics (e.g., Rothwell & Serbet, 1994; Hilton & Bateman, 2006; Bateman & Hilton, 2009). It would be particularly helpful if a much larger proportion of Mississippian spore-species could be correlated convincingly with their source plants. Improved palaeoecological interpretations would inform not only our understanding of the compositions of contrasting plant communities but also permit more precise inference of Visean climates. And confirmation of our hypothesis that the Tournaisian–early Visean pollen dates previously determined for Units 2 and 3 are incorrect would helpfully remove the most problematic anomalous data point from the Mississippian stratigraphy of the Scottish Midland Valley.

The Mississippian stratigraphy of the Scottish Midland Valley is in turn critical to solving the outstanding controversies in recent tectonic models. To quote Monaghan & Parrish (2006, p. 26), “the Midland Valley has a crucial role to play in influencing future refinements of the Carboniferous and Permian plate-tectonic model, the key being to establish if, and precisely when, extensional, sinistral strike-slip or dextral strike-slip tectonics dominated.” Most importantly, it is hypothesised that Gondwana moved northward even more rapidly than Laurasia, closing the intervening ocean obliquely from west to east and thus radically modifying climate in the equatorial belt. Warm equatorial currents formerly channelled latitudinally along the intervening ocean were consequently deflected northward, further exaggerating the polar asymmetry characteristic of the Mississippian. As the most profound period of climate change probably coincided with the acme of the Gondwana–Laurasia collision that allowed the formation of Pangaea, obtaining an accurate date for this event becomes paramount. Given that recent estimates vary substantially, ranging from the mid–late Visean (Raymond, Kelley & Lutken, 1989) to the mid-Pennsylvanian, we believe that the Kilpatrick Hills can play a yet stronger role in resolving this long-standing tectonic conundrum.

## Additional Information and Declarations

### Data Availability

The following information was supplied regarding data availability: No suitable repositories exist for the relevant categories of data.
